# Variations on a Theme: Morphological Variability and Insular Adaptations in the Pleistocene Hippopotamuses of the Siculo-Maltese Archipelago

**DOI:** 10.3390/life16081276

**Published:** 2026-07-31

**Authors:** Antonella Cinzia Marra

**Affiliations:** Department of Mathematical and Computer Sciences, Physical Sciences and Earth Sciences, University of Messina, Viale Stagno D’Alcontres 31, I-98166 Messina, Italy; antonella.marra@unime.it; Tel.: +39-090-6765452

**Keywords:** Quaternary, insular evolution, *Hippopotamus pentlandi*, *Hippopotamus melitensis*, *Hippopotamus amphibius*, *Hippopotamus antiquus*, morphometry, size reduction, Sicily, Malta

## Abstract

The Pleistocene hippopotamuses of the Siculo-Maltese Archipelago are re-examined through the study of historical collections and newly recovered material. Fossils attributed to *Hippopotamus pentlandi* and *Hippopotamus melitensis* were described anatomically and compared morphologically and morphometrically with the continental species *Hippopotamus antiquus* and *Hippopotamus amphibius*. Comparison with the continental taxa confirms the mosaic of characters that has long complicated the identification of the ancestral taxon of *Hippopotamus pentlandi*. At the same time, the Sicilian hippopotamus is shown to possess a distinctive skeletal organization, combining a shortened, more gracile skull with shortened, robust limbs. Most of the Maltese material falls within the range of *Hippopotamus pentlandi*, although it shows a general trend towards smaller size. The available record remains insufficient to determine the characters of *Hippopotamus melitensis* as a distinct endemic taxon, while the occurrence of *Hippopotamus pentlandi* in Malta cannot be excluded. The anatomical pattern documented here provides the morphological basis for future phylogenetic analyses of the Mediterranean insular hippopotamuses.

## 1. Introduction

The Pleistocene hippopotamuses of the Siculo-Maltese Archipelago provide an exceptional case study for investigating the complex interplay between morphological variability and insular evolution. Islands have long been recognized as natural laboratories where isolation, limited resources, and the absence of large predators trigger profound eco-evolutionary changes in terrestrial mammals [1]. Quaternary hippopotamids—alongside elephants and deer—frequently successfully colonized Mediterranean islands. Although their precise swimming ability and dispersal ways remain a subject of ongoing debate [2,3], their fossil record documents a widespread tendency to evolve into highly specialized endemic island forms.

In Sicily, the endemic species *Hippopotamus pentlandi* von Meyer, 1832, occurs in the late Middle Pleistocene (MIS 10-8 to MIS4) within the “Maccagnone Faunal Complex”, representing an impoverished but balanced fauna, including both moderately endemic and non-endemic species [4,5,6,7,8,9,10]. These taxa, characterized by different dispersal abilities, likely dispersed from southern peninsular Italy during a sea-level lowstand that facilitated crossing through a narrower and/or partially emergent Strait of Messina [8]. Dispersals might be related to several events that occurred during the stadial oscillations of the sea level in late Middle Pleistocene (MIS 10, MIS 8, and MIS [8]), or to a single event that occurred 0.3 Ma [8].

*Hippopotamus pentlandi* shows the lightest size reduction among the Pleistocene hippopotamuses of Mediterranean islands. In Malta, the endemic species *Hippopotamus melitensis* occurs in the Middle–Late Pleistocene within an impoverished and unbalanced insular fauna, and has been reported in association with it in selected faunal assemblages, although the mammal-based biochronological framework of the Maltese Pleistocene remains poorly resolved [1,6,9,10,11,12]. The Maltese species is considered to have evolved from *Hippopotamus pentlandi* dispersed from Sicily over the Maltese platform during sea low-stands [1,12,13,14,15]. In the re-arrangment of the Maltese faunal complexes proposed by Gatt [12], *Hippopotamus melitens* co-occurs with *Hippopotamus pentlandi* in the post-*Palaeoloxodon falconeri* Faunal Complex.

The potential ancestors of the Sicilian hippopotamus must be considered: *Hippopotamus amphibius* [11,16] and *Hippopotamus antiquus* [17,18]. Marra [10] suggested a derivation from *Hippopotamus amphibius*, attributing intermediate amphibius–antiquus characters to a possible common lineage *Hippopotamus antiquus*–*Hippopotamus amphibius*. *Hippopotamus antiquus* (including *Hippopotamus major* and *Hippopotamus tiberinus*) was the dominant European hippopotamid from the Early to Middle Pleistocene [19,20,21]. *Hippopotamus amphibius* dispersed from Africa into Europe during the Middle Pleistocene, possibly replacing *Hippopotamus. antiquus* [19,20,21], and survived in southern European refugia until the early Late Pleistocene (MIS 5 [21,22]), with debated persistence until 30–20 ka [23]. Phylogenetic relationships remain debated, with no consensus on systematics [24,25,26]. Although morphological trends support the distinction between *Hippopotamus antiquus* and *Hippopotamus amphibius*, diagnostic characters are limited and overlapping, and their relationships remain unresolved [27]. They are generally regarded as distinct taxa representing independent lineages, possibly derived from African ancestral forms [19,28,29].

Within this complex framework of the possible ancestral species, current knowledge of the Siculo-Maltese taxa is further constrained by occurrences not always referable to a clear stratigraphic context, as well as by non-systematic recoveries and museum collections that are not always reliable in terms of completeness and documentation of recovery activities.

From a historical perspective, the earliest records of *Hippopotamus pentlandi* in Sicily date to the early nineteenth century, when Scinà [30] described fossil remains from San Ciro Cave (Palermo area; see also [31]). Pentland [32] noted the similarity of the Sicilian material to the extant *Hippopotamus amphibius*, albeit at a smaller size. Von Meyer [33] subsequently assigned the Sicilian material to the species *Hippopotamus pentlandi*. Later, the rich assemblage from Cannita Cave (Palermo), studied by Accordi [34], became the main reference for the species. Subsequent studies confirmed the presence of marked variability within Sicilian hippopotamid material, interpreted as intraspecific variation or possible taxonomic heterogeneity [11,16]. Some authors proposed a subspecific status within *Hippopotamus amphibius* [35,36]. Recently, Martino et al. [37] suggested that the Sicilian record may not be taxonomically homogeneous, indicating the possible co-occurrence of *Hippopotamus amphibius* and *Hippopotamus pentlandi* in Sicily.

The biochronology of *Hippopotamus pentlandi* is constrained by its occurrence in the Maccagnone Faunal Complex (formerly the “*Elephas* [=*Palaeoloxodon*] *mnaidriensis*” Faunal Complex), which is generally assigned to the late Middle Pleistocene and possibly spans MIS 8 to MIS 4 [4,5,8]. AAR dating on *Hippopotamu pentlandi* from several Sicilian sites (San Ciro, Coste di Gigia, Puntali, Acquedolci, and Scodonì) originally yielded ages of ca. 200 ± 40 ka ([38] which were later recalculated to ca. 195 ± 39 ka for Puntali and 142 ± 36 ka for Acquedolci [39]. In addition, ESR dating of tooth enamel from the site of Contrada Fusco provided ages ranging between ca. 146.8 ± 28.7 and 88.2 ± 19.5 ka, broadly consistent with a late Middle to early Late Pleistocene age [40].

Although in some cases limited by non-systematic excavations and insufficient geological investigation, the principal hippopotamus-bearing deposits in Sicily occur in diverse depositional settings and are frequently dominated by this taxon [41,42].

In Sicily, hippopotamus-bearing deposits occur in a wide range of depositional settings. In northwestern Sicily, the classical cave localities (e.g., Maccagnone, San Ciro and Cannita) are associated with karst systems, although the fossil-bearing deposits cannot always be interpreted as primary in situ cave-floor accumulations [30,34,41,43,44,45,46]. Coastal–karst successions containing hippopotamus remains are also documented (e.g., K22, San Vito lo Capo) [47]. In northeastern and southeastern Sicily, hippopotamus-bearing deposits are associated with coastal plains, marine terrace systems, fluvio-deltaic successions, limnic deposits and coastal karst infillings, as documented at Capo Tindari, Acquedolci, Rocca Scodonì, Contrada Fusco, Coste di Gigia and Niscemi [48,49,50,51,52,53].

The Maltese paleontological record, although rich, is also affected by several limitations. Knowledge of hippopotamus occurrences in the Maltese Islands derives primarily from nineteenth and early twentieth-century investigations of cave and fissure deposits, most notably at Għar Dalam Cave [12]. Early studies mainly focused on the recovery and description of vertebrate remains, often lacking detailed sedimentological observations and precise stratigraphic control [12]. During the nineteenth century, Maltese hippopotamus remains were assigned to *Hippopotamus pentlandi* or *Hippopotamus minutus*, primarily on the basis of size [54,55]. Major [56] established *Hippopotamus melitensis* as a distinct species, smaller than *Hippopotamus pentlandi*, based primarily on material from Għar Dalam. Compared to *Hippopotamus pentlandi*, *Hippopotamus melitensis* is considered more strongly reduced in size; however, its morphology, variability, and biochronology is poorly known, as does the possible coeval or earlier presence of the Sicilian species in Malta [1,8,10,12,57]. The presence of *Hippopotamus pentlandi* on the island has been questioned by some authors, who attribute all Maltese material to *Hippopotamus melitensis* [1,58]. The faunal complexes of the Pleistocene of Malta are only tentatively defined and are attributed to dispersal events from Sicily. Ghar Dalam Cave has been intensively studied and is a reference for several cave and fissure infills across the islands, commonly associated with other elements of the endemic Pleistocene fauna [12]. These occurrences generally lack detailed stratigraphic context and independent chronological control and are often correlated with Għar Dalam on the basis of faunal composition alone. From a palaeobiogeographic perspective, the Maltese hippopotamus is generally interpreted as part of a dispersal system linking Sicily and Malta, although the taxonomic relationships and timing of colonisation remain only partly resolved [59].

The study of the hippopotamus material from both islands is limited by the nature of the historical museum collections, which largely derive from early excavations, are often poorly documented or lack secure provenance, and were affected by exchanges and donations among institutions, as commonly occurred until the mid-twentieth century (e.g., [60]). Consequently, these collections frequently consist of selected specimens that may not accurately represent the original fossil assemblages.

The present study re-examines the Pleistocene hippopotamids of the Siculo-Maltese Archipelago using the dataset originally assembled during the Author’s PhD research [61] (Supplementary Materials )expanded through the study of previously unpublished material and new anatomical observations. The principal objective is to document the morphological variation of the Sicilian and Maltese hippopotamuses and to evaluate the anatomical changes associated with insular adaptation.

## 2. Materials and Methods

The studied sample comprises remains attributed to *Hippopotamus pentlandi* and *Hippopotamus melitensis,* compared with the continental taxa *Hippopotamus amphibius* and *Hippopotamus antiquus* by direct observations and literature.

### 2.1. Provenance of Materials and State of Preservation

**Figure 1:**
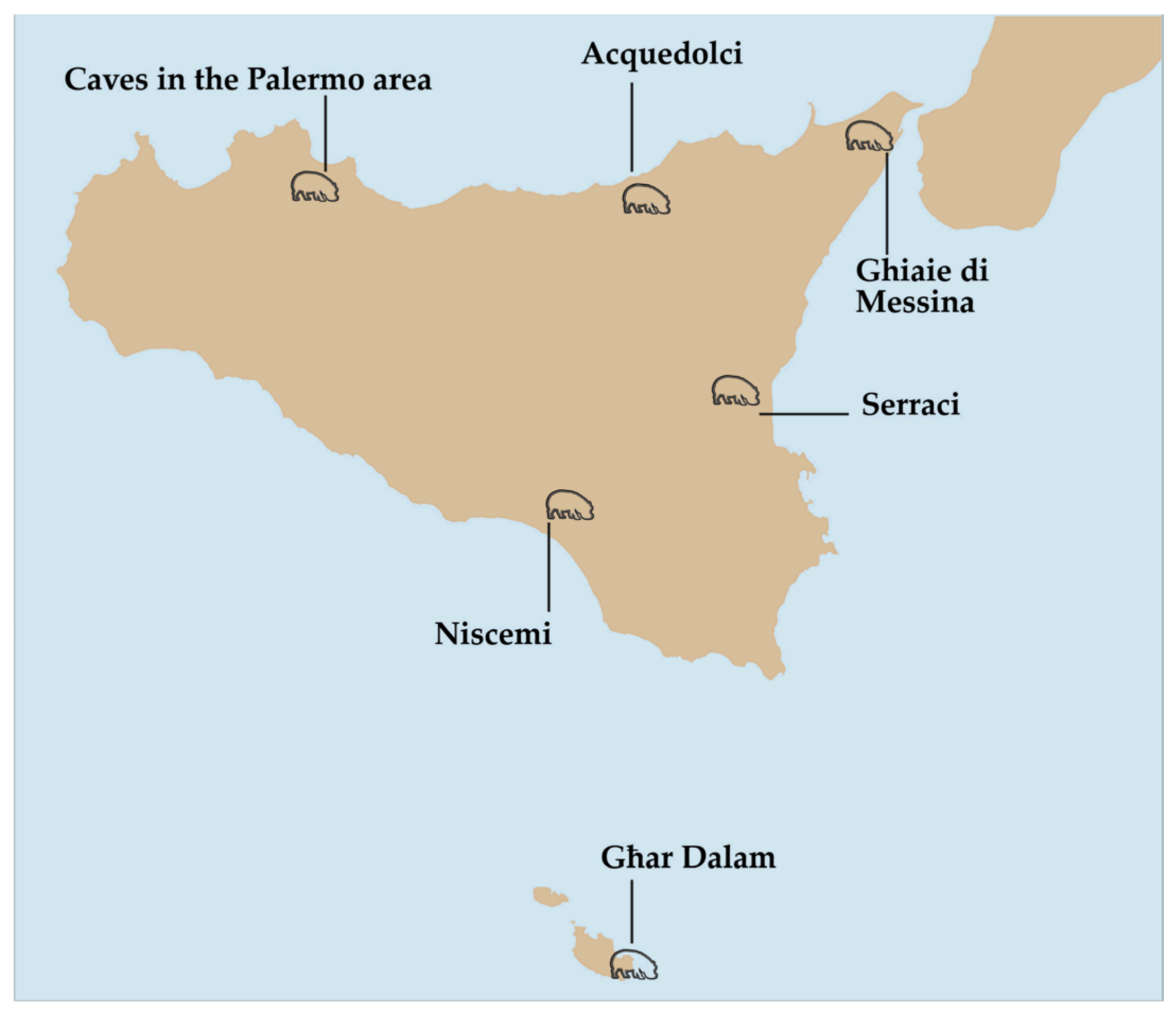
Sites of provenance of the studied materials.

The studied materials from the historical localities of Sicily, Italy, UE (Amoroso, Cannita, Maccagnone, San Ciro and Olivella caves, Palermo area, northwestern Sicily) and Malta (Għar Dalam Cave; Figure 1) are represented by museum collections (Supplementary File), which may reflect selective sampling and the subsequent dispersal of material among different institutions [12,61,62].

The hippopotamus-bearing deposits of the cave-named sites of the Palermo area are not strictly primary cave-floor accumulations. At Cannita cave, a *Hippopotamus*-dominated deposit has been reported to extend outside the cave and show evidence of strong local deformations [34,41,62]. At Maccagnone and San Ciro, the main deposits have been removed in the past and relicts of hippopotamus bone-breccia are observable outside the cave [30,31,41,43]. At San Vito lo Capo (Figure 1), a karst-cavity infill succession, starting with marine deposits and passing upward into continental levels, yields hippopotamus remains mainly from conglomerates and associated pedogenized horizons, often affected by transport and reworking (Petruso and Taschetta. At Niscemi (Figure 1), *Hippopotamus* remains derive from limnic deposits interbedded between marine units [63]. Scattered *Hippopotamus pentlandi* remains have been recovered in the “Sabbie e Ghiaie di Messina” Formation, clastic deposits interpreted as fluvial depositional systems that may be diachronous and independent, attributed to Middle Pleistocene (CARG; S1). Information on the recovery of the partially encrusted cranium, mandible and postcranials from Serraci (Figure 1) is unavailable.

The hippopotamus-dominated deposits of Acquedolci underwent a systematic excavation, which released cranial, mandibular, dental and postcranial bones [50]. The *Hippopotamus*-dominated assemblage occurs at the base of a limestone paleocliff, adjacent to a younger marine terrace (MIS 5) [50]. Amino-acid dating provided ages of ca. 200 ± 40 ka [38] later recalculated to 142 ± 36 ka [39].

The Acquedolci fossils exhibit taphonomic biases characterized by deformations and intense fragmentation with minor displacement of the fragments. Bonfiglio [50] interpreted the deposit as an in situ, low-energy lacustrine or palustrine basin formed at the base of the Pizzo Castellaro carbonate escarpment, where bone fragmentation was attributed to the post-depositional fall of boulders. However, the available trench sections and excavation photographs [50] seem to confute this classical model. The widespread association of skeletal remains with chaotic, metric-sized carbonate blocks, highly irregular substrate topography, extensive bone disarticulation, and marked lateral heterogeneity across adjacent trenches are inconsistent with a coherent, low-energy lacustrine basin. Consequently, the Acquedolci assemblage can be tentatively reinterpreted as a slope-controlled depositional complex: the *Hippopotamus pentlandi* remains originally accumulated in a wetland system situated on the elevated terrace above the escarpment; subsequent tectonic uplift and slope instability promoted repeated, short-distance gravitational mass-wasting events, redepositing and concentrating the fossiliferous sediments downslope into the sectors where they are presently exposed. Despite these limitations, the Acquedolci assemblage includes numerous cranial and mandibular remains suitable for reliable morphological assessment (Table A1 and Table A2).

The Maltese specimens included in this paper come from Għar Dalam Cave with the exception of one specimen from Melhalla. At Għar Dalam, hippopotamus remains are mainly recorded from the basal fossiliferous deposits (Layer VII), traditionally described as a bone breccia and interpreted as the principal “Hippopotamus layer” [12,43,54]. This unit documents a hippopotamus-dominated assemblage, followed by assemblages including dwarf elephants, suggesting faunal turnover and possibly multiple dispersal events ([12] and references therein). Sedimentological observations indicate that the basal fossiliferous layer represents an unsorted and unbedded accumulation, interpreted as a mass-flow deposit rather than a primary in situ assemblage [12]. Hippopotamus remains are also reported from the overlying red earth deposits (Layers V–IV), although their stratigraphic significance remains uncertain due to possible reworking [12]. The taphonomic context at Għar Dalam suggests that fossil accumulation may reflect a combination of processes, including natural mortality, sediment transport, and reworking within cave and fissure systems [55,56].

### 2.2. Methods

Morphological descriptions and measurement protocols follow Mazza [19]. The study includes crania, mandibles, teeth, and long bones. Among the autopodial elements, only the astragalus was considered because of its diagnostic value and comparability. Specimens clearly displaying the described characters are indicated for cranii and mandibles characters only; long-bone descriptions are presented without specimen-by-specimen citations. The studied fossils are listed in Supplementary Materials. Literature-derived measurements were integrated into the dataset and explicitly referenced. Comparative observations were based on direct examination of cranial material of *Hippopotamus antiquus* and *Hippopotamus amphibius* (Supplementary Materials), supplemented by published descriptions where needed.

Preservation state, completeness, and ontogenetic stage, relevant to character assessment, were systematically considered and are summarized in Appendix A, Table A1 and Table A2 for cranial and mandibular specimens. Ontogenetic stages of mandibular specimens were assigned according to Laws [64]. All studied long bones belong to adult individuals with completely fused epiphyses. Morphometric variation was analyzed using scatterplots and Simpson’s ratio diagrams, using Microsoft Excel (Office 365).

**Figure 2:**
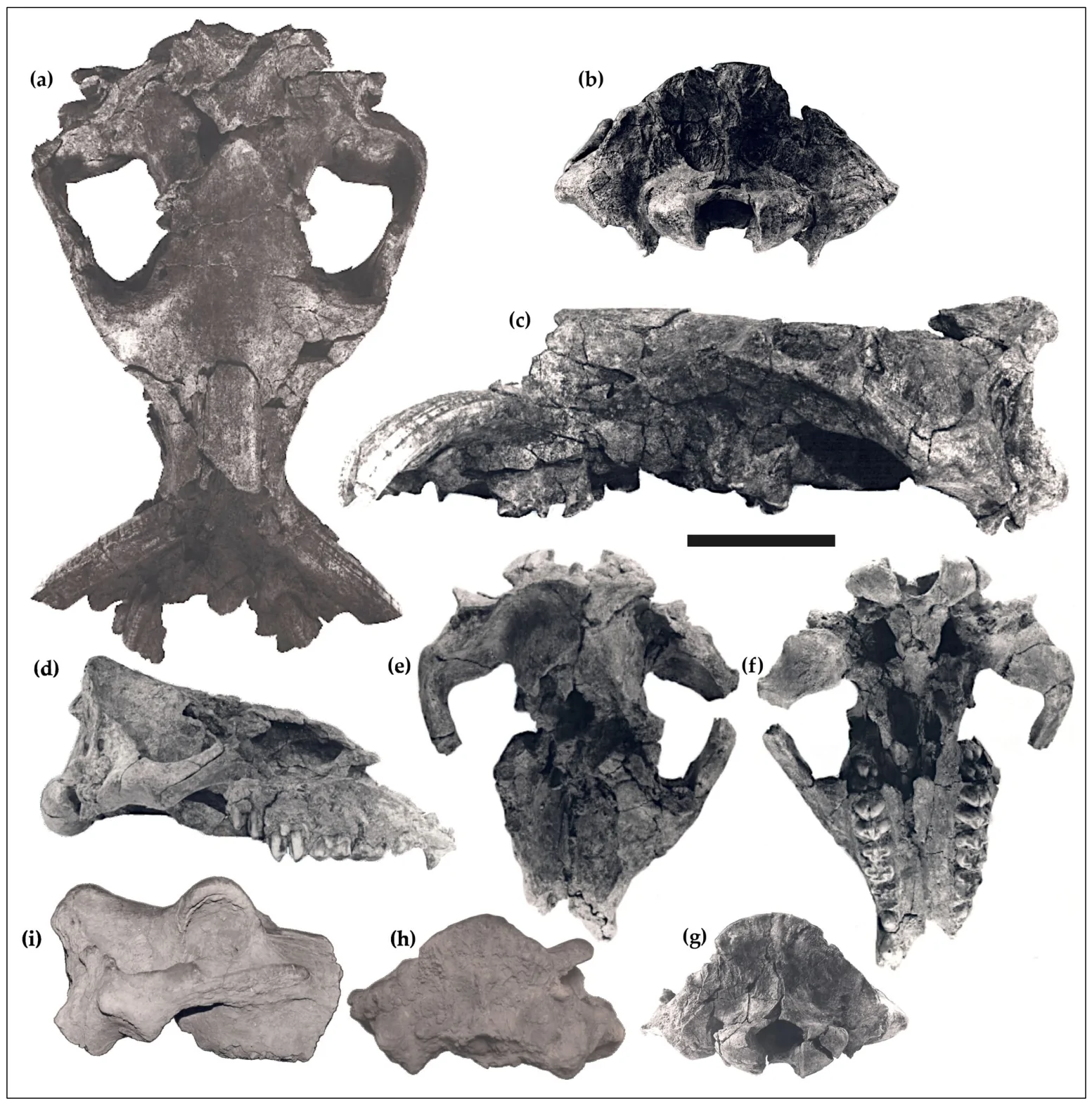
Crania of *Hippopotamus pentlandi*: CrAcq1 in (**a**) dorsal, (**b**) lateral, and (**c**) occipital views; CrAcq5 in (**d**) lateral, (**e**) dorsal, (**f**) ventral, and (**g**) occipital views; CrSer1 in (**i**) lateral and (**h**) occipital views; modified after [61] scale bar: 10 cm.

**Figure 3:**
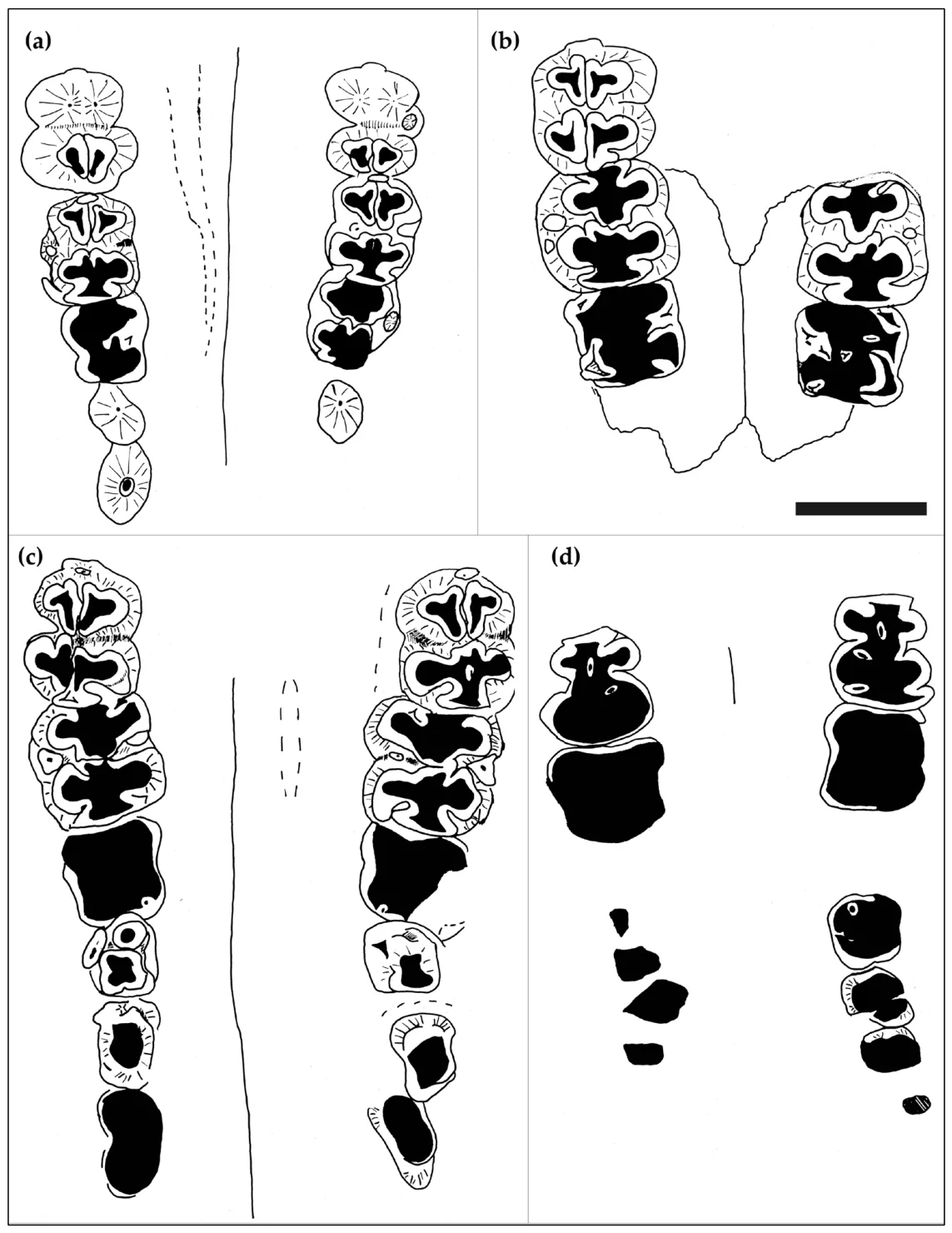
Occlusal area of upper dentition at different ontogenetic stages, from the youngest to oldest: (**a**) tooth raw preserving M2-M1, D4, P3, P2, CrAcq1; (**b**) tooth raw M3-M1, CrAcq2; (**c**) tooth raw M3-P2, CrAcq3; (**d**) tooth raw M3-M2, P4-P2, CrAcq4. Scale bar: 5 cm. Drawings by the Author.

**Figure 4:**
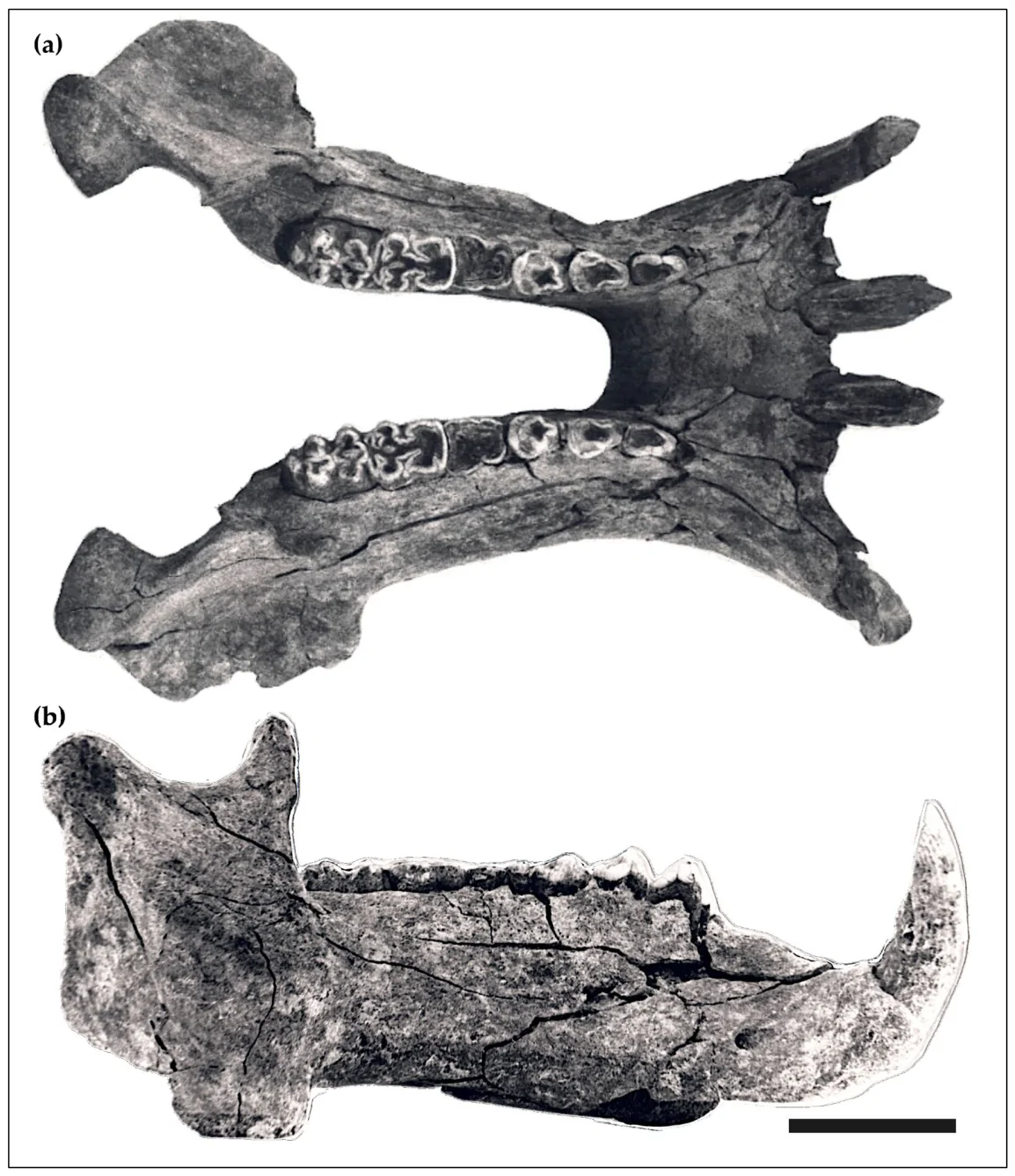
Mandible of *Hippopotamus pentlandi:* (MandAcq3) in (**a**) dorsal view and in (**b**) lateral view; modified after [61] scale bar: 5 cm.

**Figure 5:**
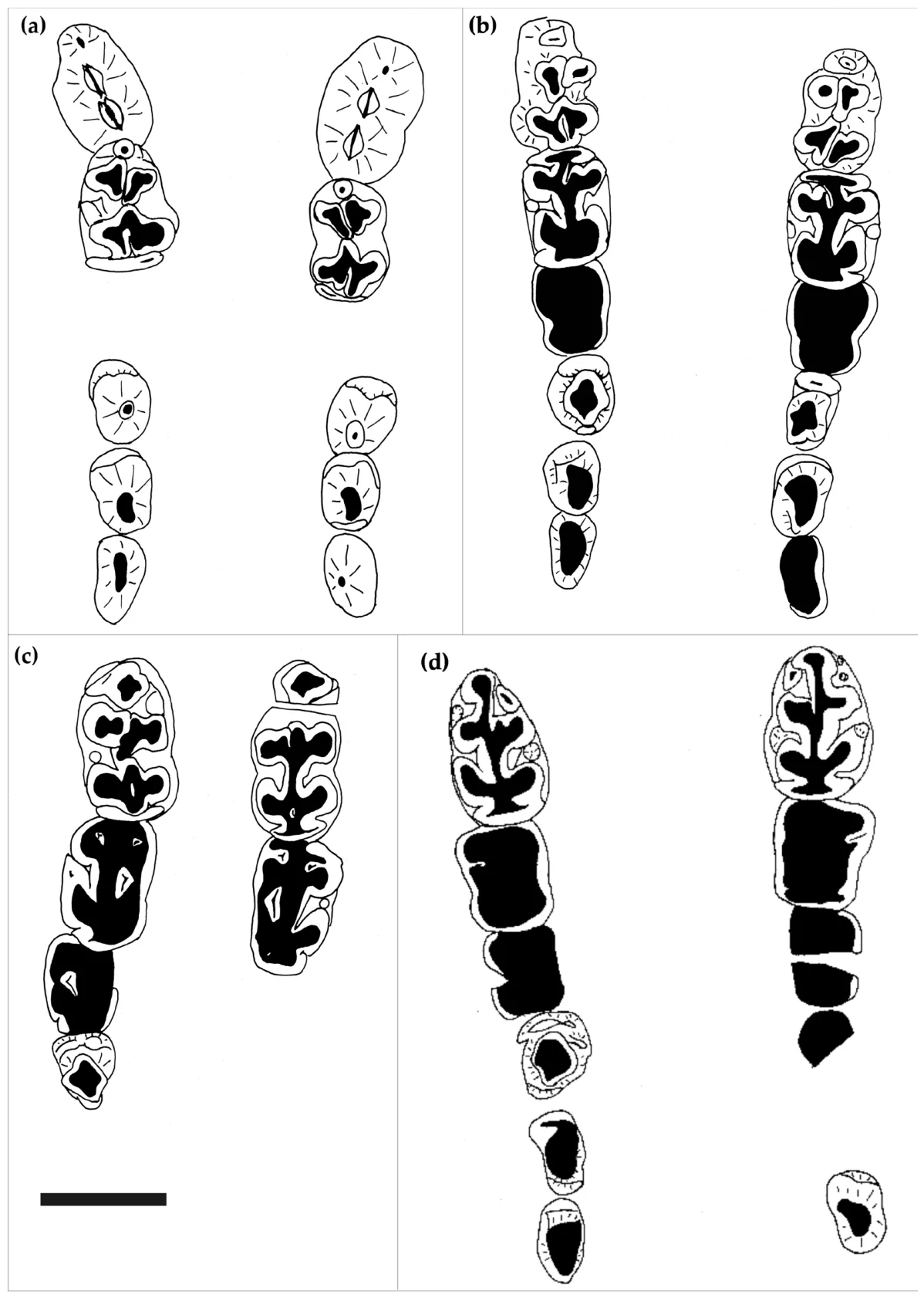
Occlusal area of lower dentition at different ontogenetic stages, from the youngest to oldest (wear stages and related ontogenetic age according to [64]): (**a**) MandAcq2 (Stage XI, 20 +/− 3 years old. (**b**) MandAcq3 (Stage XIV, 27 +/− 3 years old). (**c**) MandAcq4 (Stage XVI, about 33 +/− 3 years old. (**d**) MandAcq5 (Stage XVII, about 35 +/− 4 years old). Scale bar: 5 cm. Drawings by the Author.

**Figure 6:**
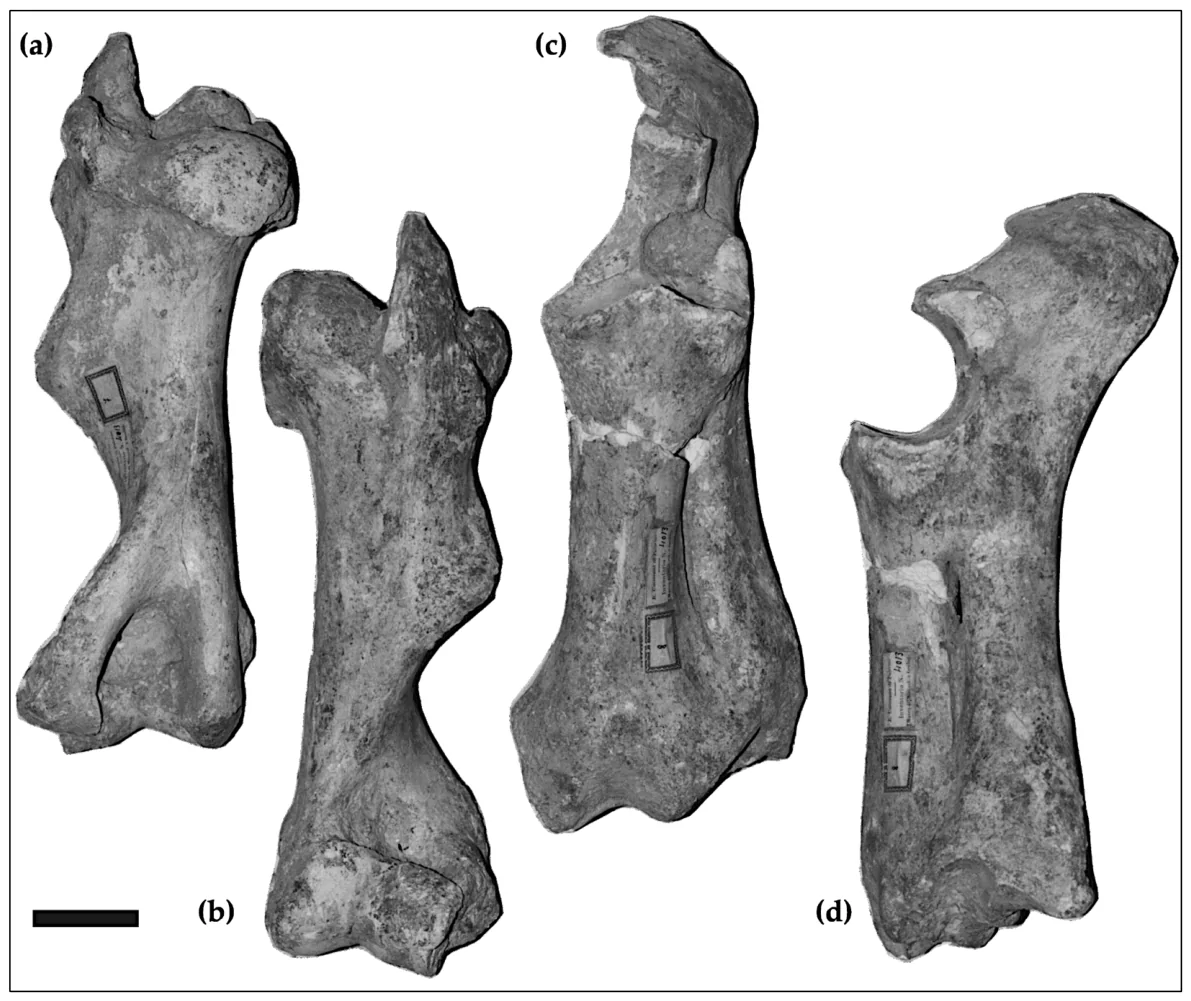
Forelimb bones of *Hippopotamus pentlandi*. Humerus (OmCan5) in (**a**) anterior and (**b**) posterior views. Radius-Ulna (RuCan5) in (**c**) anterior and (**d**) medial views; modified after [61] scale bar: 5 cm.

**Figure 7:**
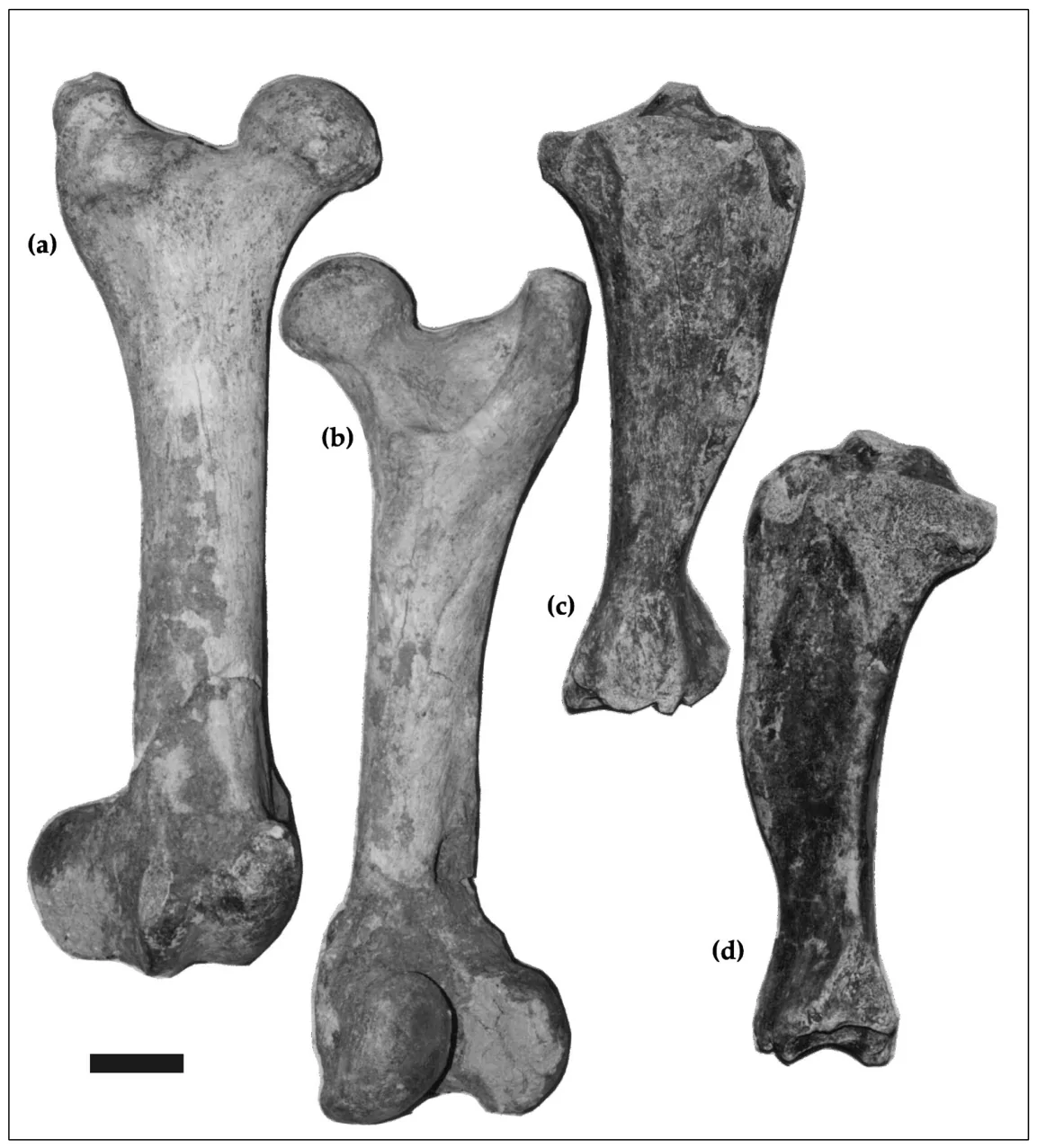
Femur (FemCan2) of *Hippopotamus pentlandi* in (**a**) anterior and (**b**) posterior views. Tibia (TiOli1) in (**c**) anterior and (**d**) lateral views; modified after [61] scale bar: 5 cm.

**Figure 8:**
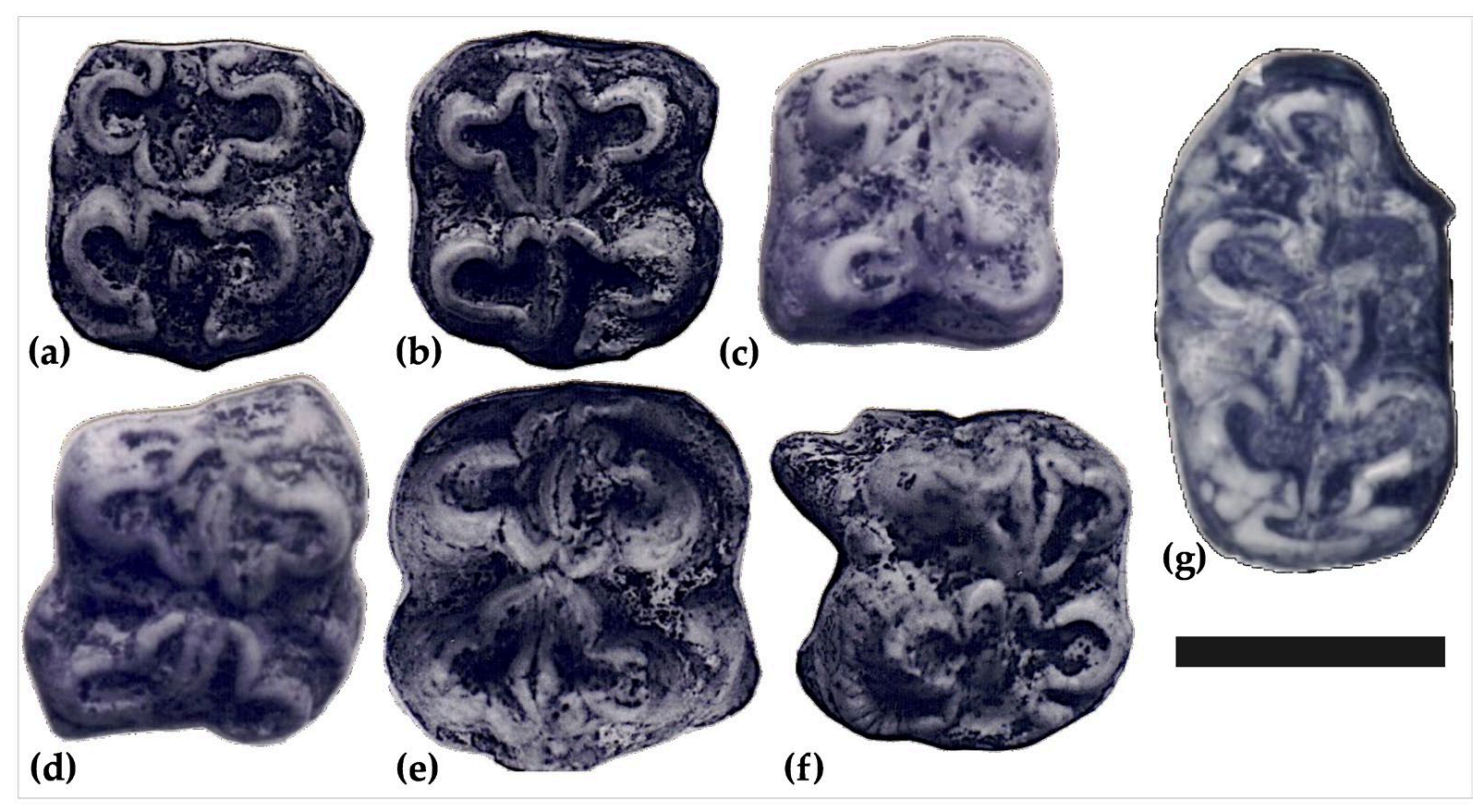
Molars of *Hippopotamus melitensis* in occlusal view: (**a**) M1/GhD3; (**b**) M1/GhD2; (**c**) M1/GhD5, (**d**) M2/GhD8, (**e**) M2/GhD2, (**f**) M2/GhD8, (**g**) m3/GhD6; modified after [61] scale bar: 5 cm.

Figure 2, Figure 3, Figure 4, Figure 5, Figure 6, Figure 7 and Figure 8 were prepared using photo editing software (GIMP 2.10.38). Figure 3 and Figure 5 were hand-drawn using transparent acetate sheets placed over the specimens and a fine-tipped marker. The illustrations were subsequently transferred onto tracing paper, inked, scanned, and assembled using GIMP.

## 3. Systematic Paleontology

Order: Artiodactyla Owen, 1848.

Family: Hippopotamidae Gray, 1821.

Genus *Hippopotamus* Linnaeus, 1758.

*Hippopotamus pentlandi* Von Meyer, 1832.

### 3.1. Morphological Description of Hippopotamus pentlandi

#### 3.1.1. Cranium

Dorsal view (Figure 2a,e). The external occipital protuberance forms a flattened to slightly rugose triangular area, from which the sagittal crest extends anteriorly; this crest is often badly preserved and can be evaluated mainly at its basis, where it may be narrow and elevated (specimen CrAcq3; CrCan8) or broad and blunt (CrAcq1, CrAcq5, CrSc17, CrSer1). Temporal lines diverge markedly from the sagittal line, although in one subadult specimen (CrAcq1) they are less laterally divergent and more anteriorly directed, possibly indicating ontogenetic influence. The braincase is small, inflated, and short antero-posteriorly. The frontal bone is broad, posteriorly concave, and anteriorly flat. Nasals are elevated relative to the maxillae, which are laterally flat and slightly depressed (CrAcq5). The zygomatic arch is laterally compressed and moderately narrow, joining the rostrum through an oblique transition; the zygomatic bone merges with the maxilla without forming a sharp angle (CrAcq1, CrAcq5, CrSer1). The rostral constriction is short. The rostrum expansion is observable in only one specimen (CrAcq5), where it is not wider than the zygomatic arches.

Lateral view (Figure 2c,d,i). The occipital squama is subvertical, bearing a nuchal crest and projecting condyles (CrAcq1, CrAcq 5, CrCan8, CrSc17, CrSer1). The sagittal crest gently slopes anteriorly (CrAcq1, CrAcq 5, CrCan8, CrSc17, CrSer1). Orbits are elevated and robust (CrSer1). The zygomatic arch shows a longitudinal depression on its ventrolateral surface and is divided by a ridge into a dorsolateral and a ventral component; this ridge continues onto the maxilla (CrAcq5, CrSer1). The infraorbital foramen is positioned at the level of P4. The nasal profile is rising gently from the suture with the frontal bone to the level of the fourth premolar and then descending anteriorly (CrAcq3, CrAcq5).

Occipital view (Figure 2b,g,h). The occipital bone is subtriangular in on subadult specimen (CrAcq1) and becomes trapezoidal in adults due to slight widening of the mastoid margin and rounding of the parietal profile. The occipital squama is high relative to mastoid width. The nuchal and lateral tuberosities are well developed. Occipital condyles are conical with a horizontal major axis (CrAncq5, CrCan8), while appears oblique in CrSc17, probably a subadult from the shape of the occipital. The foramen magnum varies from sub-quadrangular (juvenile specimen, CrAcq1 Figure 2d,f) to sub-rectangular with a major horizontal axis (adults, Figure 2a–c,h,i).

Ventral view (Figure 2f). The basioccipital is robust, with well-developed tuberosities (CrAcq1, CrAcq5, CrSc17). The choanae are located posterior to M3. Glenoid cavities are weakly concave. The retroarticular process is broad and well developed. The palate is narrow, often flat, with slight constriction at M1–P4 (CrAcq1, CrAcq3, CrAcq4, CrAcq5, CrCan3). The dental arches are straight and anteriorly convergent in the subadult specimen CrAcq1, becoming slightly convergent at the level of M1 in adults (CrAcq3, CrAcq4, CrAcq5, CrCan3).

#### 3.1.2. Upper Dentition

M3. Occlusal outline rectangular, with rounded margins and a posterior talon smaller than the anterior one. The cingulum is more pronounced in the labial side, where a labial interlobar column may occur. In slightly worn specimens, the posterior cones are lower than the anterior ones and the labial cones are curved lingually. Wear progressively produces the typical trefoil pattern starting from the anterior cones (Figure 3b,c). In old individuals, only a peripheral enamel rim is preserved (Figure 3d).

M2. In slightly worn specimens, the cusps are well preserved and separated by deep grooves (Figure 3a). The talons are broader than in M3, the anterior talon being slightly more developed than the posterior. The cingulum is robust and a labial interlobar column is usually present. With increasing wear, the cones acquire the typical trefoil pattern and progressively enlarge, while the talons become increasingly indented through contact with adjacent teeth (Figure 3b,c). In advanced wear stages, the occlusal outline becomes squared (Figure 3d).

M1. Smaller than M2 and M3 and characterized by relatively thin enamel. Slightly worn specimens are uncommon in the sample studied and present a finely striated enamel. Wear first affects the anterior cones (Figure 3a). In advanced wear stages, the posterior talon becomes deeply indented through contact with M2, whereas the anterior margin generally remains straight or slightly dented and weared by the contact with P4 (Figure 3 b,c).

P4. Occlusal outline ranging from subquadrate with rounded margins to nearly circular. The tooth is massive, with thick enamel and a robust cingulum. In slightly worn specimens, the main cusp is curved lingually and posteriorly and bears four grooves. A strong postero-lingual accessory column is present. The neck is high and thick, forming two triangular talons. As ontogenetic growth advances, the progressive rotation of the tooth repositions the talons anteriorly and posteriorly (Figure 3b–d), the cusp section changes from oval to cruciform, whereas the accessory column becomes more circular. In advanced wear stages, both structures fuse into a broad subcircular wear surface and the cingulum is progressively reduced (Figure 3d).

P3. The tooth is suboval and broader posteriorly, with a laterally compressed cusp and thick enamel, finely striated. In slightly worn specimens (Figure 3a), the cusp bears three crests (anterior, posterior, and lingual). The cingulum is particularly robust on the labial side and forms anterior and posterior talons, the latter strongly developed, with small digitations and two robust columns. Wear broadens the cusp and accentuates its anteroposterior elongation, whereas the posterior talon is progressively reduced (Figure 3c). In heavily worn specimens, enamel is preserved only along the margins (Figure 3d).

P2. The tooth is oval and constricted at mid-length. The cusp is strongly compressed laterally. In slightly worn specimens, it shows a concave posterior face, a straighter anterior face, and two crests (labial and lingual, Figure 3a). The cingulum is higher on the labial side and particularly robust posteriorly, where it is digitated. With wear, the cusp acquires a longitudinally oval outline and retains an occlusal surface raised centrally and sloped anteriorly and posteriorly (Figure 3c,d).

C. Strongly curved and triangular in cross-section, with a deep groove running along the lower surface. Wear produces an oblique facet directed towards the outer side.

I2. Curved downwards, with an oval cross-section elongated anteroposteriorly. The enamel forms alternating raised and depressed bands, particularly on the anterior surface. The wear facet is deeply concave and directed posteriorly.

I1. Directed downwards, with a rounded cross-section. The wear facet is concave.

#### 3.1.3. Mandible

Dorsal view (Figure 4a). The mandibular condyles have a broad and slightly convex articular surface, almost flat (Mand Acq3, MandCan1). The horizontal rami slightly diverge posteriorly (MandAcq3, MandAmo1, MandSer1). The external surface of the mandible slopes outward as it descends (MandAcq3, MandCan1, MandAmo1, MandSer1). The mandibular symphysis is deep and concave in adults (MandAcq3, MandSc7-11). The dental arches are straight and slightly convergent at the level of the first molar–fourth premolar (MandAcq2, MandAcq3, MandAcq5, MandAmo1).

Lateral view (Figure 4b). The horizontal ramus is high relative to the vertical ramus and reaches its maximum height at the level of the premolars (MandAcq3, MandCan1, MandSer1). The coronoid-condylar notch is widely concave, with a thin margin. The coronoid process is thin, directed upward and inward, and has a straight anterior margin (MandAcq3, MandCan1, MandSer1). The posterior profile of the vertical ramus is almost straight, with a not protruding gonion caudalis. The masseteric fossa is deep. The posteroventral border of the ascending ramus shows a slight convexity (MandAcq3, MandCan1, MandSer1). The anterior border of the mandibular process forms an almost right angle with the inferior border of the horizontal ramus (MandAcq3, MandCan1, MandSer1). The inferior profile of the horizontal ramus ranges from slightly convex to flat and to slightly concave, and its lateral surface is slightly concave (MandAcq3, MandCan1, MandSer1). The posterior mental foramen opens toward the third–fourth premolar, while the anterior mental foramen opens toward the alveolus of the first premolar.

Posterior view. The two mandibular rami diverge markedly downward and converge upward (MandAcq3, MandAmo1). The medial surface of the ascending rami is flat and bounded anteriorly by a ridge extending toward the third molar. Anterior to this ridge, the rami remain subparallel, whereas posterior to it they diverge markedly. The anterior margin of the mandibular foramen aligns with the anteromedial margin of the coronoid process. The mandibular condyles have their major axis passing through the apex, directed inward and downward (MandAcq3, MandAmo1).

#### 3.1.4. Lower Dentition

m3. Strong posterior tubercle and weak cingulum. Accessory columns may occur between the posterior tubercle and the endoconid or hypoconid. The endoconid often shows a reduced or absent posterior stylid, producing a comma-shaped to subcircular outline (Figure 5b,d). Wear produces the typical trefoil pattern; in advanced stages the conids fuse with the talonids and posterior tubercle (Figure 5d).

m2. Rectangular, with well-developed anterior and posterior talonids, weak or absent cingulum, and usually a labial interlobar column. The endoconid may show a comma-shaped outline. Wear produces a trefoil pattern and progressively fuses conids and talonids (Figure 5a,b); in advanced stages the tooth is shortened by talonid wear and indention with m3 and m1 (Figure 5d).

m1. Rectangular and smaller than m2, with thinner enamel. Morphology generally comparable to m2. In advanced wear, the anterior and posterior margins are indented by contact with p4 and m2 (Figure 5b–d).

p4. Massive premolar with thick enamel, robust cingulum, and subcircular outline elongated anteroposteriorly. The main cusp bears four strong crests, producing a cruciform wear pattern (Figure 5b,c). The posterior talonid is strongly developed; with wear, cusp and talonids broaden (Figure 5d).

p3. Oval premolar elongated anteroposteriorly and slightly broader posteriorly (Figure 5a,b,d). Cingulum robust, especially labially, sometimes with accessory columns. Posterior talonid more developed than the anterior one. Wear may produce a centrally raised occlusal surface sloping anteriorly and posteriorly.

p2. Oval, anteroposteriorly elongated, and constricted at mid-length (Figure 5a,b,d). Posterior talonid more developed than the anterior one. In slightly worn specimens, the cusp is curved lingually.

c. Lower canine robust and triangular in cross-section, with subparallel longitudinal enamel grooves and a broad groove along the anterointernal surface.

Incisors. Incisors straight and directed upwards. I2 has an oval cross-section and subvertical wear facet; I1 bears a lateral wear facet, sometimes forming a step.

#### 3.1.5. Long Bones of the Forelimb

Humerus (Figure 6a,b).

Proximal view. The bicipital groove is deep and narrow. The crest of the greater tubercle is well defined by a raised margin bordering a flattened or slightly depressed area; it is slightly rugose and subcircular in shape. This feature seems to be age-related and appear more extensive in some specimens. The summit of the greater tubercle is robust, arched, and directed inward, with a uniformly concave inner margin. The lesser tubercle is sub-oval with a flat surface. The anterior articular facet is flat and transversely extended.

Cranial view. The deltoid tuberosity is broad and prominent, positioned laterally and supported by a robust, slightly arched humeral crest that projects toward the lateral plane. The *teres major* tubercle, visible cranially and medially, is elongated and only slightly prominent, appearing as a roughened area. The trochlea is high and uniformly convex. The transverse axis of the trochlea slopes downward toward the lateral plane. The sagittal crest on the lateral condyle is pronounced. The groove between condyle and trochlea is deep. The epitrochlea does not project beyond the trochlear profile.

Lateral view. The tricipital curved line is sharp and robust at its base, extending toward the neck in a straight course.

Caudal view. The articular head is broad with slight convexity, more developed posteriorly. The neck is distinct. The *teres minor* tubercle is thin and raised. The olecranon fossa is deep, triangular in outline, and narrower superiorly. The epicondylar crest is straight, massive, and flattened. The coronoid fossa is wide and deep. A flattened facet is present, inclined between the horizontal and lateral planes. In lateral view, the epicondylar crest gives a straight caudal profile, connected with a constriction above the distal end.

Radius–Ulna (Figure 6c,d).

Cranial and lateral views. The bone is flattened antero-posteriorly and appears thin in lateral view. The olecranon is slender, directed backward, with a squared margin; its beak points outward and downward, projecting prominently. The summit of the olecranon is directed medially, squared in shape, robust, thick, and rugose. The medial surface of the olecranon is uniformly concave, whereas the lateral surface is convex. The cranial margin is concave. The greater sigmoid notch is deep and regularly circular in profile. The synovial fossa is deep. The proximal articular fossae of the radius are nearly equal in size: the medial fossa is uniformly concave, whereas the lateral fossa is concave near the saddle separating the two fossae and flatter toward the medial margin. The coronoid process is robust, pointed in some specimens and blunt in others. The bicipital tuberosity and lateral radial tuberosity are well developed. The radial styloid process is robust and rounded. The groove for the *extensor carpi radialis* is confined to the articular area and does not extend onto the epiphysis, which is swollen near the articular surface. A pronounced roughness is present on the lateral surface of the ulna near the lateral radial tuberosity; in juveniles this appears as a swelling. The radio-ulnar groove is deep and opens into a small interosseous arch. The dorsal margin of the radius is nearly straight. The ulnar styloid process is not particularly prominent, whereas the radial styloid process is broad and pronounced, with blunt margins.

Caudal and medial views. The caudal margin of the ulna is broad, blunt, and uniformly concave. The medial surface of the radius and ulna is concave. The distal articular facets show moderate concavities and saddles.

#### 3.1.6. Long Bones of the Hind Limb (Figure 7)

Femur (Figure 7a,b).

Cranial and lateral views. The summit of the greater trochanter is robust and located approximately at the same height as the head. The trochanteric crest is strong and blunt, descending to end slightly above the inferior margin of the lesser trochanter. The convexity of the trochanter is rough. The trochanteric notch is short and deep. The lateral surface of the trochanter is flat and rough, as extensive as the trochanteric crest that runs alongside it. The head does not rise above the greater trochanter. The neck is broad, short, and well defined, only slightly projecting outward. The profile from the neck to the trochanter is slightly concave in some specimens, but in most cases it is straight. The lateral supracondylar crest is robust and continuous with a crest extending to the proximal extremity. The lateral lip of the trochlea is small and blunt. The medial lip of the trochlea is broad with a rounded profile, and the adjacent medial surface is slightly concave.

Caudal and medial views. The neck is well delineated. The intertrochanteric crest, straight or slightly concave, runs obliquely from the summit of the trochanter toward the lesser trochanter. The intertrochanteric fossa is broad and deep, ranging from triangular to oval in cross-section. The lesser trochanter is slightly swollen, projecting, and mildly rough. The insertion area for the *iliacus* and *psoas major* is rough. The medial surface is flattened below the lesser trochanter. A thin raised crest begins near the base of the trochanter and runs along the lateral margin of the bone, becoming broader and more rounded before reaching the lateral supracondylar crest. The shaft is robust. The supracondylar fossa is deep, especially superiorly, and circular in cross-section. Its longitudinal axis is oblique relative to the shaft axis. The intercondylar fossa is very deep and narrow, with its axis inclined toward the lateral margin. The medial condyle is broad and projecting. The lateral condyle is rounded, with an articular facet facing toward the centre and showing a broad concavity; the facet itself is nearly flat. The lateral epicondyle is swollen and projecting, with a flattened facet. The trochlea shows marked torsion toward the medial side.

Tibia (Figure 7c,d).

Cranial and lateral views. The central intercondylar area is deep and narrow. The tibial spine is raised, prominent, and robust, with a broad base. The lateral tuberosity is strong and projecting. The tibial tuberosity is robust, broad, and prominent. The tibial groove is wide and shallow. The tibial crest is strong and projecting, especially at mid-shaft. The medial malleolus is prominent and robust. The fibular notch is wide, shallow, and triangular.

Caudal and medial views. The popliteal line is narrow and curved. The medial tuberosity is robust. The proximal articular facets are relatively flat, whereas the distal ones are deeply concave.

#### 3.1.7. Astragalus

Proximal view. The trochlea exhibits an external margin more developed than the internal one, which posteriorly extends into a tubercle of variable development. The degree of development of the external margin shows notable individual variability.

Anterior view. The proximal articular surface extends onto the anterior face to nearly half the length of the bone, where it is interrupted by a deep horizontal groove. In some specimens, a vertically elongated fossa is present at the centre of this groove. Below this groove lies the anterior portion of the distal articular surface, subdivided into two facets by a ridge of variable prominence, oriented toward the internal side.

Distal view. The external distal facet is convex, whereas the internal one is concave near the ridge and convex toward the internal margin of the bone.

Posterior view. The posterior articular surface for the calcaneum is slightly concave and bounded by a broad external groove and another groove extending along the internal and superior sides.

Lateral view. The anterosuperior external facet is broad, flat, and crescent-shaped. The other two external facets, located more posteriorly, are sub oval.

Order: Artiodactyla Owen, 1848.

Suborder: Whippomorpha Spaulding et al., 2009.

Family: Hippopotamidae Gray, 1821.

Genus: Hippopotamus Linnaeus, 1758.

Species: *Hippopotamus melitensis* Major, 1902.

Type locality: Għar Dalam Cave, Malta.

Geological range: Middle–Late Pleistocene.

### 3.2. Morphological Descriptions of Hippopotamus melitensis

#### 3.2.1. Molars

Upper M3. The anterior talon is robust and broad. The paracone is small, with wide anterior and posterior grooves. The anterior parastyle is short, whereas the posterior one is longer than the posterior protostyle. The protocone is rounded and larger than the paracone, with narrow anterior and posterior grooves. The anterior protostyle is longer than the anterior parastyle, while the posterior protostyle, although long, is shorter than the posterior parastyle. The metacone is small, with wide anterior and posterior grooves. Both anterior and posterior metastyles are short. The hypocone is small, with wide grooves. The anterior hypostyle is longer than the metastyle and directed toward the lingual side, whereas the posterior hypostyle is very short. The posterior talon is weak. A labial interlobar column is present. The cingulum is robust, especially around the anterior cusps.

Upper M2 (Figure 8d–f). The paracone is rounded, with narrow anterior and posterior grooves. The anterior parastyle is short and rounded, whereas the posterior one is longer. The protocone is rounded, with wide anterior and posterior grooves. The anterior protostyle is longer than the anterior parastyle, whereas the posterior protostyle is shorter than the posterior parastyle. The metacone shows a wide anterior groove and a narrow posterior one. The anterior metastyle is long and directed toward the lingual side, whereas the posterior metastyle is shorter. The hypocone shows a wide anterior groove and a narrow posterior one. The anterior hypostyle is long, whereas the posterior one is short. Both anterior and posterior talons are robust; the posterior one is narrower than the anterior and has a tubercle-like shape. A robust labial interlobar column is present. The cingulum is very robust on the labial side.

Upper M1 (Figure 8a–c). Most molars are at a moderate stage of wear, so they belong to young specimens. The interlobar column is not always present and, when present, is not particularly robust. The paracone is rounded, with short styles. The posterior groove is narrow, whereas the anterior one is wider. The protocone has an anterior style slightly longer than the parastyle; grooves are wide. Posterior cusps have anterior styles longer than the posterior ones. No crossing between styles is observed.

Upper P3. The cusp has an oval cross-section. The cingulum is strong.

Lower m3 (Figure 8g). The central crossing between stylids is well developed. The anterior endostylid is long and directed toward the labial side, whereas the posterior endostylid is absent. The anterior hypostylid is weakly developed, while the posterior one is long. The posterior metastylid is elongated, crosses the anterior hypostylid, and is directed toward the lingual side. The posterior protostylid is short. The two anterior stylids are generally similar in development. The posterior tubercle is robust. The cingulum is well developed. In some molars, the cement is very abundant.

Lower p4. The cingulum is robust anteriorly. A lingual column is present. The cusp is cruciform in shape.

Lower p3 (or p2). The cingulum is weakly developed. Wear is moderate and sloping, being higher in the central portion and decreasing toward the anterior and posterior margins.

#### 3.2.2. Tibia

The preserved distal end of the tibia shows strongly concave distal articular facets and a hook-shaped medial malleolus directed toward the articular surface. The two specimens are small if compared to *Hippopotamus pentlandi*. The larger specimen is characterized by a shallow central intercondylar area, a weakly developed and rounded tibial spine, and a shallow distal articular surface. The fibular notch is triangular and flat, and the proximal articular surfaces are weakly convex. The smaller specimen shows a deep and narrow intercondylar area, a prominent and robust tibial spine, and deeply concave distal articular facets. The fibular notch is wider and shallow, and the proximal articular facets are relatively flat. The two specimens show differences in the depth of the intercondylar area, the development of the tibial spine, and the morphology of the distal articular surface (shallow vs. deeply concave).

## 4. Comparisons

### 4.1. Cranium

Compared with *Hippopotamus antiquus*, in dorsal view *Hippopotamus pentlandi* shows a generally smaller external occipital protuberance, although a juvenile specimen exhibits a more extensive and flatter occipital area and a broader sagittal crest, approaching the condition observed in *Hippopotamus antiquus*. The sagittal crest is variable and exhibits features recalling both *Hippopotamus antiquus* and *Hippopotamus amphibius*, but it is always shorter than in *Hippopotamus antiquus*. The frontal concavity and neurocranium length appear intermediate between *Hippopotamus antiquus* and *Hippopotamus amphibius*. The zygomatic arch is laterally compressed and moderately narrow, and the zygomatic breadth is reduced relative to both continental species. The muzzle is comparable to that of *Hippopotamus amphibius* and shorter than in *Hippopotamus antiquus*. In adult specimens, the rounded parietal profile resembles the condition observed in *Hippopotamus amphibius*, whereas the occipital condyles appear more horizontally oriented than in *Hippopotamus antiquus*.

In occipital view, the subadult specimen of *Hippopotamus pentlandi* has a generally subtriangular outline, whereas adults show a semicircular outline due to a slight widening of the mastoid margin and rounding of the parietal profile, as seen in *Hippopotamus amphibius*. The condyles are robust and oriented even more horizontally than in the continental species *Hippopotamus antiquus*.

In ventral view, the choanae of adult specimens are positioned posterior to the third molar, as in *Hippopotamus antiquus*. The glenoid cavities appear broader both laterally and anteroposteriorly than in the available comparative material of *Hippopotamus antiquus* and *Hippopotamus amphibius*. The palate is short, as in *Hippopotamus amphibius*, but differs due to a slight convergence at the M1–P4 level and a weak divergence of the premolar row, features that are present and more pronounced in *Hippopotamus antiquus*. The diastema between the second and third premolars, which occurs in *Hippopotamus antiquus* and, to a lesser extent, in *Hippopotamus amphibius*, is absent in *Hippopotamus pentlandi*.

**Figure 9:**
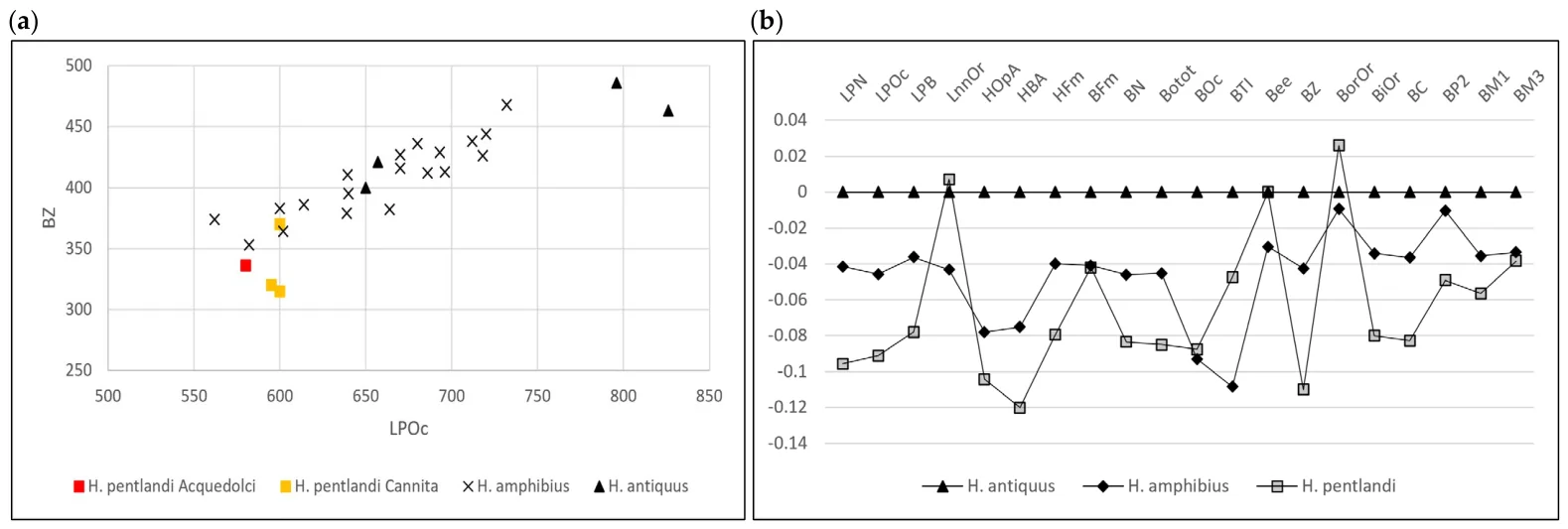
Metric comparisons of the crania: (**a**) scatterplot of LPOc (prosthionoccipital condyle length) vs. BZ (Zygomatic Breadth); (**b**) Simpson’s ratio diagram of the measurements, baseline: *Hippopotamus antiquus*.

The metric comparisons are affected by the small number of specimens, which, however, to date represent the largest available dataset for the species. The scatterplot of the prosthion-occipital condyle length (LPOc) vs. the zygomatic breadth (BZ) confirms the relative reduction in BZ in *Hippopotamus pentlandi*, although one specimen from Cannita falls within the variability range of *Hippopotamus amphibius* (Figure 9a).

The Simpson’s log-ratio diagram is calculated on very few specimens, so it must be interpreted with caution (Figure 9b). The skull length is consistently reduced with respect to the continental baseline species (*Hippopotamus antiquus*). However, a relative elongation of the LPB (prosthion-basion length) is observable, likely due to a backward displacement of the basion, which is related to the sturdiness of the basioccipital bone. In the rostral region, LnnOr is problematic due to the bias induced by the high fragmentation of specimens CrAcq 3 and CrAcq 5 (Table A1).

The occipital heights HOpA (opisthion-acrocranion height) and HBA (basion-acrocranion height) are smaller than in the continental species, and the proportional reduction in HBA is probably related to the relative values of HOpA, due to the low position of the opisthion. This feature is also evidenced by the elevated breadth of the foramen magnum, related to a low height (HFm), concordant with the observed rectangular shape. BN (breadth of the nuchal crest) and Botot (breadth across otion) are proportionally smaller than in the continental species, whereas the observed condyles larger than *Hippopotamus amphibius* are revealed by the proportionally lower reduction in BOc (breadth across the occipital condyles). The proportional increase in BTI (breadth across the temporal lines) compared to *Hippopotamus amphibius* reflects the observed development of the temporal lines, intermediate between the continental species. The low lateral extension of the zygomatic arch is confirmed by the strong reduction in BZ (zygomatic breadth). The position of Bee (euryion-euryon breadth) reflects the observed swollen braincase but is probably over-dimensioned due to the preservation status of CrAcq 3 and CrAcq 5 (Table A1), which reliably caused the outlier datum of Boror (breadth between the orbital cavities). This bias is indirectly proved by its position within the general reduction range of BiOr (infraorbital breadth). This latter datum, along with BC (breadth across the canine alveoli) and BP2 (breadth between the second premolar alveoli), shows a proportional reduction confirming a rostrum narrower than in the continental species. The observed constriction of the palate at the first molars is confirmed by the reduction in BM1 (breadth between the first molar alveoli), followed by a wide palate at the third molars, where BM3 (breadth between the third molar alveoli) approaches *Hippopotamus amphibius*.

### 4.2. Mandible

In dorsal view, the mandibular branches of *Hippopotamus pentlandi* diverge more than in *Hippopotamus antiquus* and less than in *Hippopotamus amphibius*. The tooth rows are slightly convergent at the first molar–fourth premolar level (m1–p4). This convergence is marked in *Hippopotamus antiquus* and almost absent in *Hippopotamus amphibius*.

In lateral view, the mandible is relatively low, as in *Hippopotamus antiquus*, but shorter. The lower outline of the horizontal branch is concave and the margin is robust, as in *Hippopotamus amphibius*. The height of the ascending branch appears intermediate between *Hippopotamus antiquus*, where it is lower, and *Hippopotamus amphibius*, where it is higher. The coronoid process is proportionally thinner than in the available comparative material of the mainland species. The sigmoid notch is wide and rounded. The masseteric fossa is wide and deep, resembling the condition observed in *Hippopotamus amphibius*. The posterior outline is relatively straight, as in *Hippopotamus antiquus*, lacking the protruding gonion caudalis observed in *Hippopotamus amphibius*.

**Figure 10:**
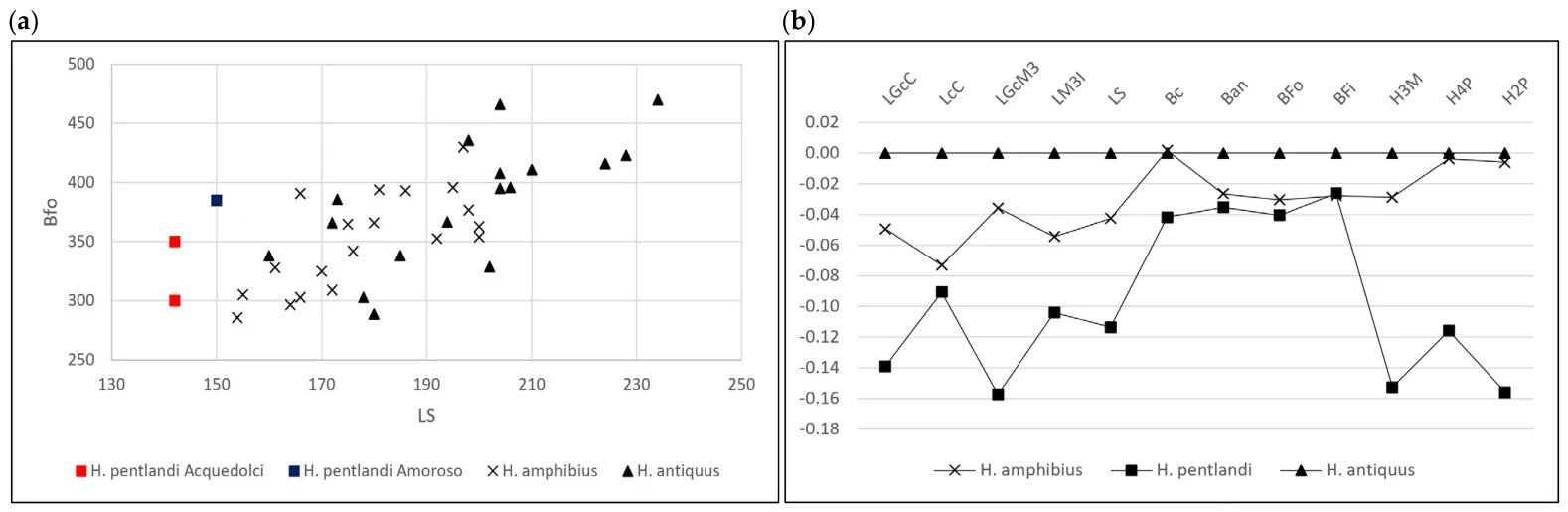
Metric comparisons of the mandibles: (**a**) scatterplot of Bfo (outer breadth of the rostral fan) vs. LS (length of the mandibular symphysis); (**b**) Simpson’s ratio diagram of the mandible measurements, baseline: *Hippopotamus antiquus*.

The measurements of the mandible are incomplete and render the application of linear morphometrics not fully reliable. Although the scatterplot comparing BFo (outer breadth of the rostral fan) and LS (length of the mandibular symphysis) is applicable to only three specimens, it has been included for the successive discussion (Figure 10a). The Sicilian specimens fall outside the variability of the continental species, displaying a markedly short symphysis. The considered characters also show high variability within the continental species themselves.

The Simpson’s ratio diagrams (Figure 10b) show a marked reduction in LGcC (gonion caudalis-canine alveolus length) and LGcM3 (gonion caudalis-posterior border of m3 length), consistent with the observed morphology of the straight posterior margin. The length between the mandibular condyle and the canine alveolus (LcC) and the length between the posterior border of the m3 alveolus and the incisor alveolus (LM3I) are less reduced, possibly because these measures do not include the posterior border. The symphysis length (LS) is markedly shorter than in the continental species.

The breadths (Ban: breadth of the two halves across the condyles; BFo: outer breadth of the rostral fan; BFi: inner breadth of the rostral fan) are proportionally less reduced and close to *Hippopotamus amphibius*, with Bc (breadth across the condyles) slightly reduced, which is possibly related to the occipital morphology. The extremely marked reduction in H3M (height of the horizontal ramus at the level of m3) is related to the concavity observed in the lower border of the mandible. The lower margin follows a straighter profile anteriorly at the p4 and p2 levels (H4P, H2P).

### 4.3. Dentition

In general, the molars of *Hippopotamus pentlandi* are smaller than those of *Hippopotamus antiquus* and *Hippopotamus amphibius* and slightly differ in proportions. The molars of *Hippopotamus melitensis* are smaller than those of *Hippopotamus pentlandi*, without significant morphological differences. The third lower molar of the insular species, particularly the Maltese form, often presents a “comma-shaped” entoconid. This wear pattern, (sensu Mazza [19]), derives from the reduced development or complete absence of the posterior style of the cusp and may occur in both continental and insular species. Comparisons focus on the upper and lower third molars, which are clearly identifiable when isolated and allow extended comparisons with published metrics.

The tooth eruption sequence of the upper permanent dentition of *Hippopotamus pentlandi* (Figure 3) is comparable to that of extant *Hippopotamus amphibius* (=C, M1 → P2 → P3, M2 → M3 → P4, according to [19,65]). The lower permanent dentition (Figure 4) also appears comparable to the tooth eruption sequence of extant *Hippopotamus amphibius* (c → m1 → p2 → p3, M2 → p4 → m3, according to [19,65]). The lack of maxillary and mandibular remains belonging to the same individuals does not allow confirmation that odontogenesis is consistently accelerated in the lower jaw, although this can be considered a common trait of hippopotamids.

The molars of *Hippopotamus melitensis*, although morphologically similar to those of *Hippopotamus pentlandi*, are smaller and show the frequent development of “comma-shaped” entoconids and the reduction or absence of posterior styles. Compared to continental species, they appear shorter and proportionally broader. In addition, the posterior talon, particularly in M3, appears relatively weakly developed, and the interlobar column in M1 is not consistently present and, when present, is only weakly expressed. In some specimens, the cement is very abundant and may form thick deposits associated with strongly developed cingula and columnar digitations.

The upper third molar (M3) of *Hippopotamus pentlandi* closely follows the general pattern observed in *Hippopotamus antiquus* and *Hippopotamus amphibius*. All taxa share a trefoil wear pattern, progressive fusion of styles and talons, and an anteroposterior wear sequence. Differences are expressed mainly through size and proportions. In *Hippopotamus pentlandi*, the M3 is generally smaller and more compact, with a quadrangular outline, a relatively reduced posterior talon and a robust cingulum.

**Figure 11:**
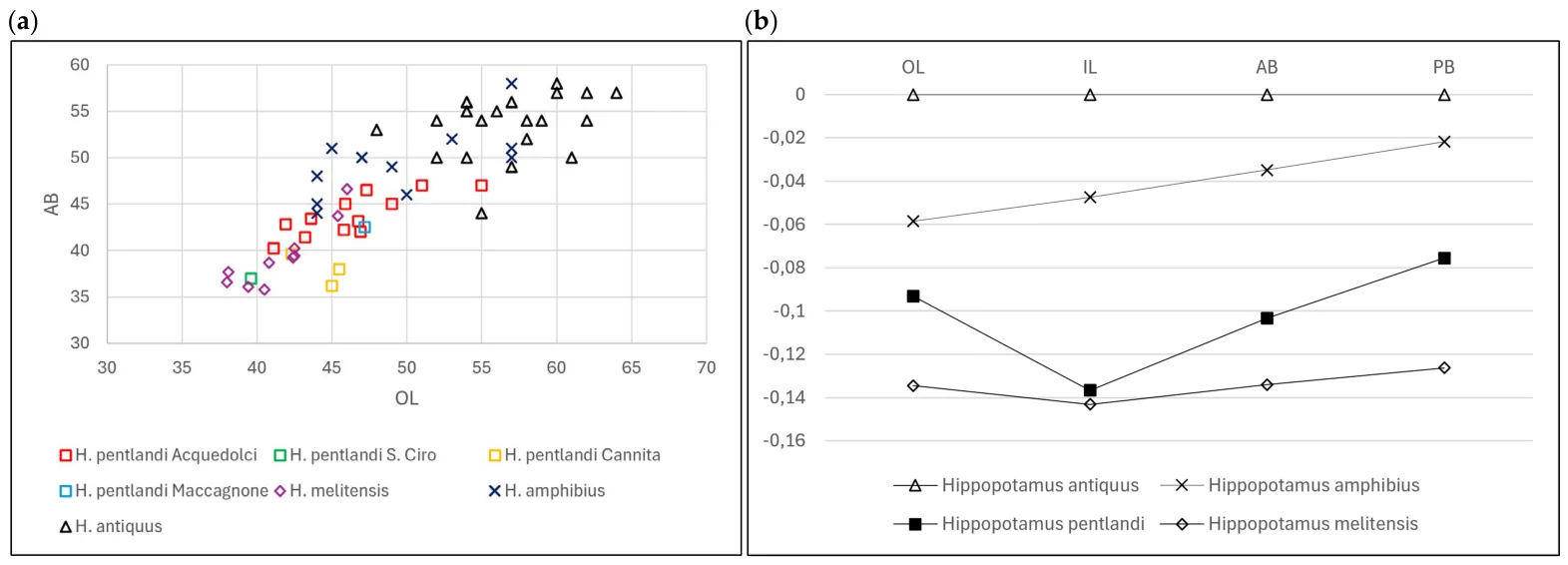
Metric comparisons of the third upper molars: (**a**) scatterplot of AB (anterior breadth) vs. OL (outer length); (**b**) Simpson’s ratio diagram of the molar measurements, baseline: *Hippopotamus antiquus*.

The upper third molar shows not only size-related variation (Figure 11a), with *Hippopotamus antiquus* occupying the largest values, *Hippopotamus amphibius* and *Hippopotamus pentlandi* an intermediate range, and *Hippopotamus melitensis* the smallest. Among the Sicilian samples, Acquedolci shows the widest range, including both large and small specimens, whereas Cannita and San Ciro plot at lower values; Maccagnone falls within the Acquedolci range. The largest specimens of *Hippopotamus melitensis* overlap the Sicilian species. The scatterplot also highlights a clear change in proportions of the insular species, confirming a third molar more “squared”. In the Simpson ratio diagram, the reduction of the lingual side (IL) is marked in *Hippopotamus pentlandi*, while it is slight in *Hippopotamus melitensis*.

**Figure 12:**
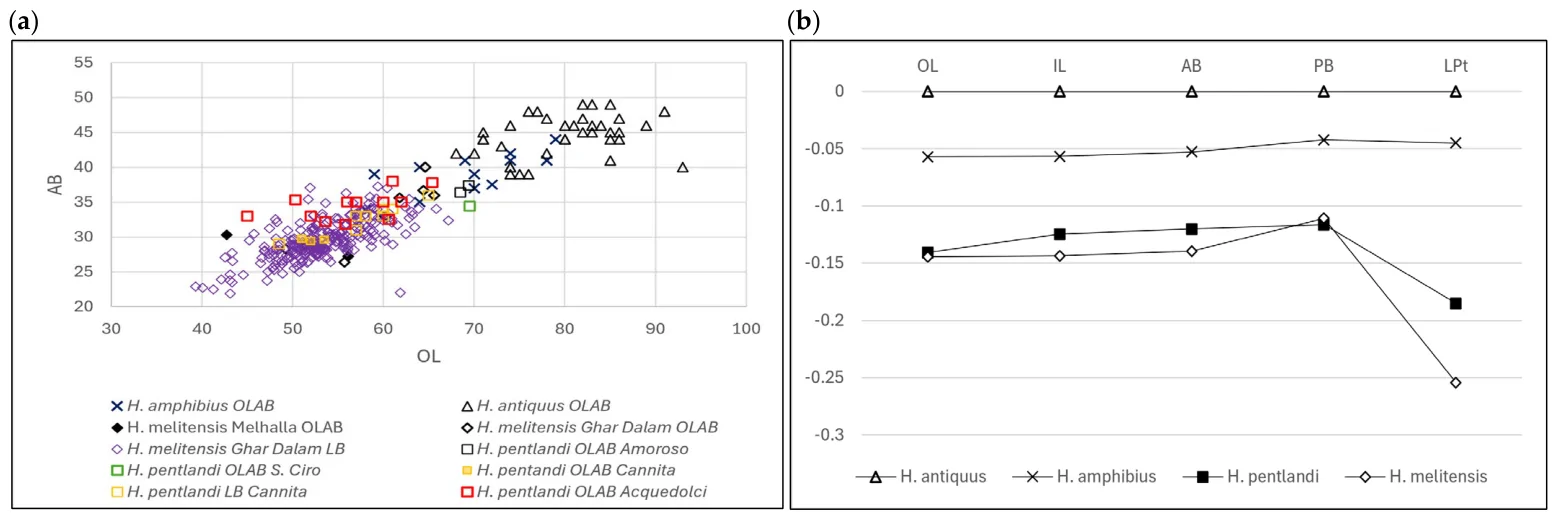
Metric comparisons of the third lower molars: (**a**) scatterplot of AB (anterior breadth) vs. OL (outer length); (**b**) Simpson’s ratio diagram of the molar measurements, baseline: *Hippopotamus antiquus*.

The lower third molar appears conservative in its overall proportions, showing a proportional and continuous trend towards the small size of the insular species (Figure 12a). Acquedolci displays the widest range of variation among the Sicilian samples, whereas Cannita plots at lower values; the San Ciro and Maccagnone specimens fall within the Acquedolci range, while Amoroso specimens (form one mandible) are very large. The conspicuous sample from Għar Dalam overlaps the lower variability observed in *Hippopotamus pentlandi* and define a smaller cluster of few elements. The scatterplot includes specimens measured as maximum length and breadth (L and B), considered equivalent to outer length (OL) and anterior breadth (AB).

In the Simpson’s ratio diagram (Figure 12b), the continental species show differences mainly in the dimensions, whereas *Hippopotamus pentlandi* shows reduction in the outer length (OL), proportional increase in the posterior breadth and very marked reduction in the posterior tubercle (LPt). *Hippopotamus melitensis* has a very similar pattern, with a more marked posterior enlargement (PB) and a more extreme reduction in the posterior tubercle (LPt).

### 4.4. Long Bones of the Forelimb

#### 4.4.1. Humerus

The humerus of *Hippopotamus pentlandi* is very robust, sturdier than *Hippopotamus antiquus* and *Hippopotamus amphibius*. Some morphological characters are similar to *Hippopotamus antiquus*: the summit of the greater tubercle directed medially; a straight epicondylar crest; a weakly projecting epitrochlea; and a triangular olecranon fossa. The well-developed deltoid tuberosity resembles that of *Hippopotamus amphibius* but is more extended longitudinally than in the continental species.

**Figure 13:**
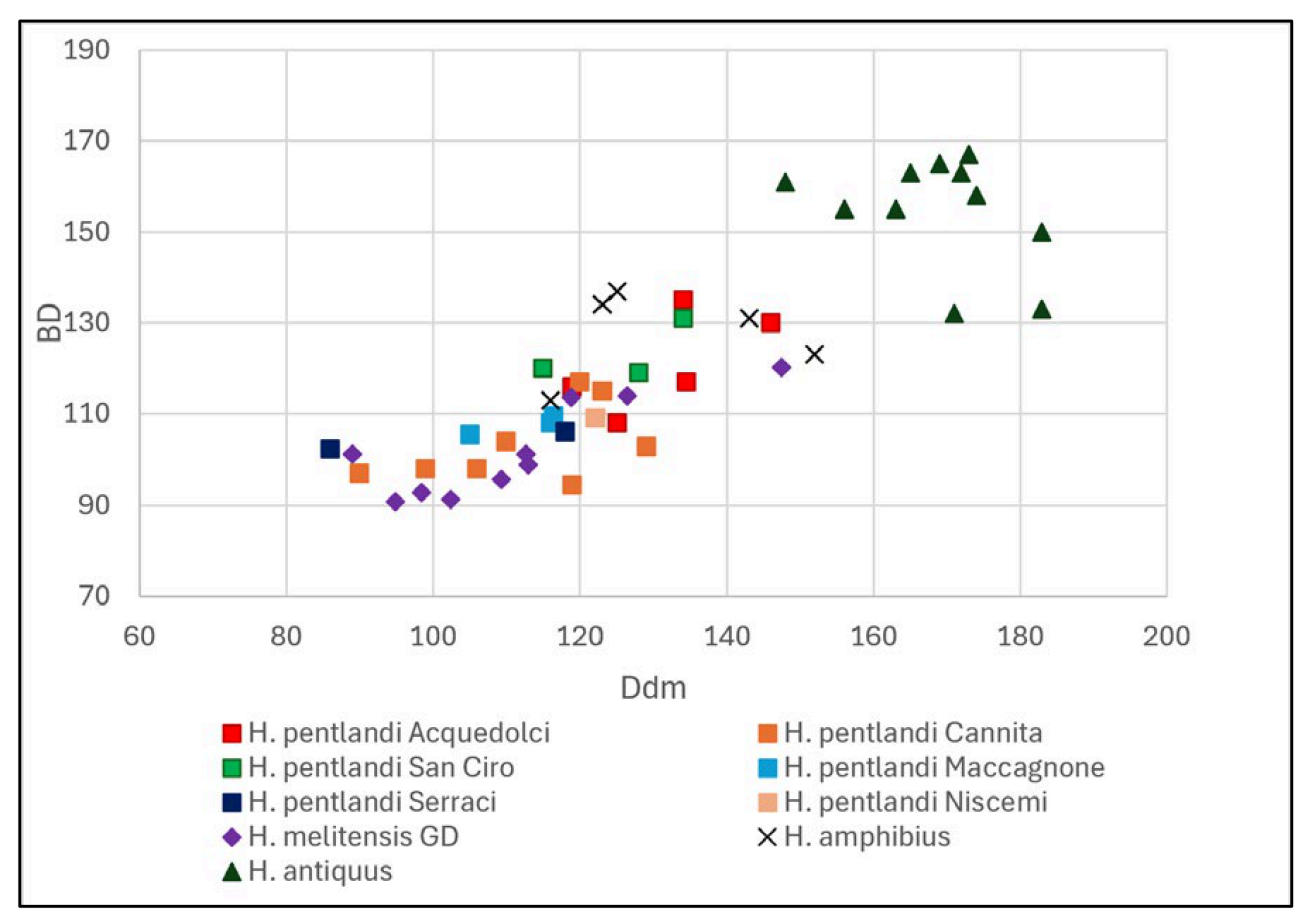
scatterplot of BD (distal breadth) vs. Ddm (distal depth on the medial side) of the humerus.

In the scatterplot of distal breadth (BD) versus distal depth on the medial side (Ddm) of the humerus (Figure 13), *Hippopotamus antiquus* is the largest specie and shows a comparatively slenderer distal articulation. *Hippopotamus amphibius* shows intermediate dimensions and proportions. *Hippopotamus pentlandi* and *Hippopotamus melitensis* are in the lower range, reflecting their overall reduced size, and are characterized by a relatively broader distal articulation compared to the more ‘gracile’ *Hippopotamus antiquus*. An extended overlap between *Hippopotamus pentlandi* and *Hippopotamus amphibius* is observable.

**Figure 14:**
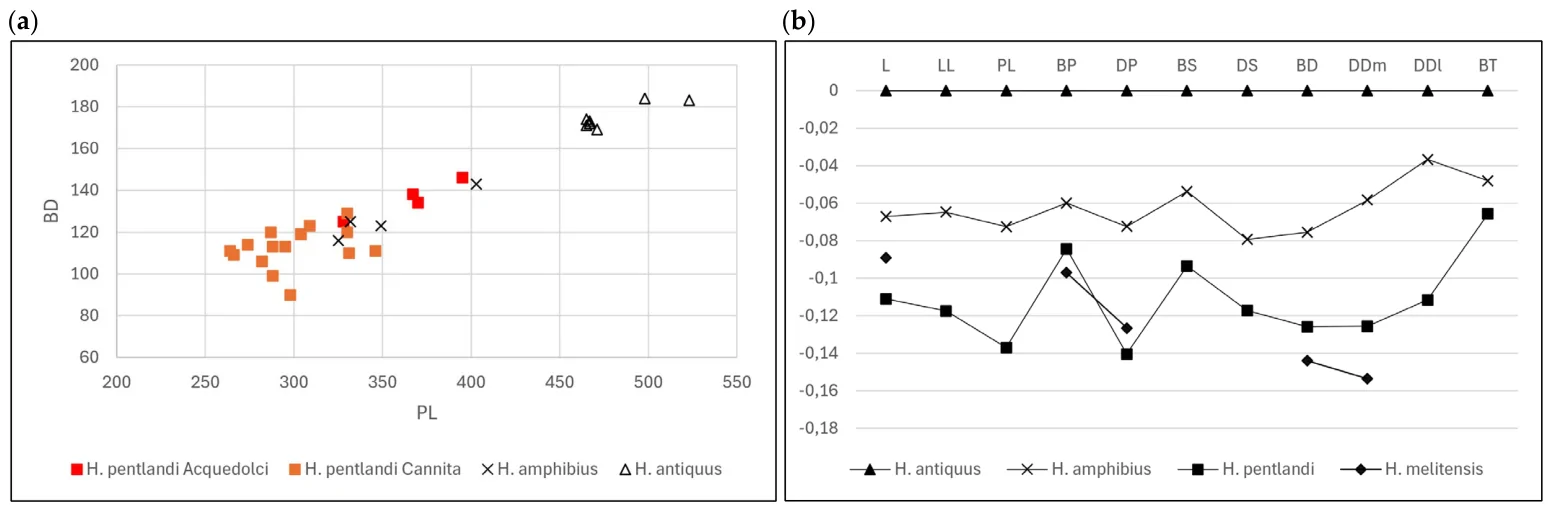
Metric comparisons of the humerus: (**a**) scatterplot of BD (distal breadth) vs. PL (physiologic length); (**b**) Simpson’s ratio diagram of the humerus measurements, baseline: *Hippopotamus antiquus*.

The scatterplot of distal breadth (BD) compared to the physiologic length (PL) indicates overlap of *Hippopotamus pentlandi* with *Hippopotamus amphibius* for the specimens from Acquedolci and for the larger specimens from Cannita Cave, with a distal breadth (BD) proportionally larger (Figure 14a). The Simpson ratio diagram (Figure 14b) of *Hippopotamus pentlandi* show lengths significantly below the continental species. The physiological length (PL) has a more pronounced reduction in the total (L) and lateral (LL) length, probably the relative projection of the greater tubercle compared to the lesser tubercle, rather than a uniform scaling of the entire humerus. The proximal end is sturdy in breadth (BD) but very compressed in depth (DP).

The distal end appears almost proportionally reduced in size with respect to *Hippopotamus amphibius*, but with a distal breadth of the trochlea (BT) approaching this species. Considering that the distal breadth (BD) is significantly reduced, the trochlea occupies a large part of the distal breadth and the epitrochlea is reduced.

#### 4.4.2. Radius-Ulna

The radius–ulna does not show highly distinctive characters. *Hippopotamus pentlandi* has a robust, squared, and fairly vertical olecranon, resulting in a sigmoid notch facing more laterally than in *Hippopotamus amphibius*.

**Figure 15:**
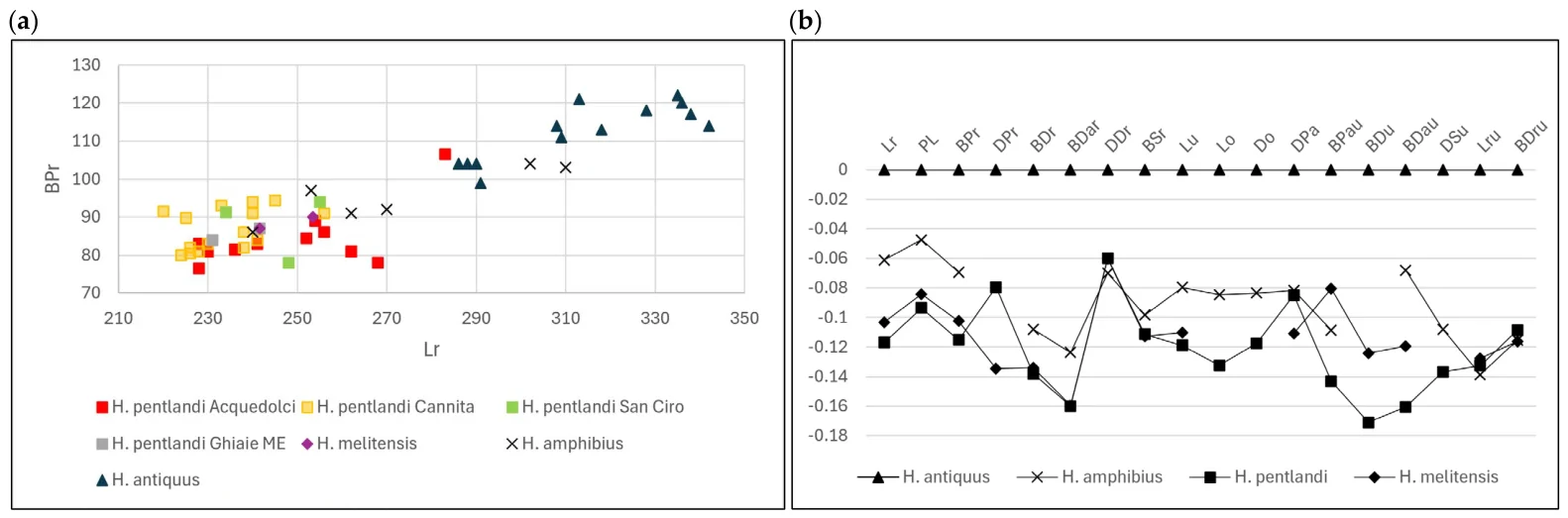
Metric comparisons of the radius-ulna: (**a**) scatterplot of BPr (proximal breadth of the radius) vs. Lr (radius length); (**b**) Simpson’s ratio diagram of the radius-ulna measurements, baseline: *Hippopotamus antiquus*.

The scatterplot of the radius length (Lr) vs. the proximal radial breadth (BPr) shows that *Hippopotamus pentlandi* is partially overlapping with *Hippopotamus amphibius*, whereas underwent a significant reduction in physiological length (Lr) compared to *Hippopotamus antiquus*, with the proximal radial breadth (BPr) remaining very large, maintaining a high degree of robusticity (Figure 15a). This is consistent with the observed morphology. *Hippopotamus melitensis* falls within the range of *Hippopotamus pentlandi* and has a comparable distal end).

In the Simpson’s ratio diagrams, the significant negative values of lengths of *Hippopotamus pentlandi* (Lr, Lu, Lru, Lo, Lu) indicate the shortening of the forelimb (Figure 15b). The breadth of the proximal epiphysis (BDr) of radius is not strongly reduced, while the breadth of the articular surface of radius (BDar) is. The radius epiphysis (DDr) and the anconeal process have a relevant depth, near *Hippopotamus amphibius*. The shaft of *Hippopotamus pentlandi* is robust (BSr), approaching *Hippopotamus amphibius*, as the very strong distal epiphysis breadth (BDRu). The limited *Hippopotamus melitensis* specimens show a trend of dimensional and proportional reduction rather congruent with *Hippopotamus pentlandi*.

#### 4.4.3. Femur

The femur of *Hippopotamus pentlandi* shows the head and neck more vertically oriented than in *Hippopotamus amphibius*, being more similarly oriented than *Hippopotamus antiquus*, although in the latter the neck is markedly longer than in the Sicilian remains. The fairly straight profile from the head to the greater trochanter, the robust shaft, the projecting lateral epicondyle, and the deep, oblique supracondylar fossa are similar to those observed in *Hippopotamus antiquus*, whereas the weak rotation of the trochlea resembles that of *Hippopotamus amphibius*. The of the proximal and distal epiphyses appear massive and with a pronounced.

**Figure 16:**
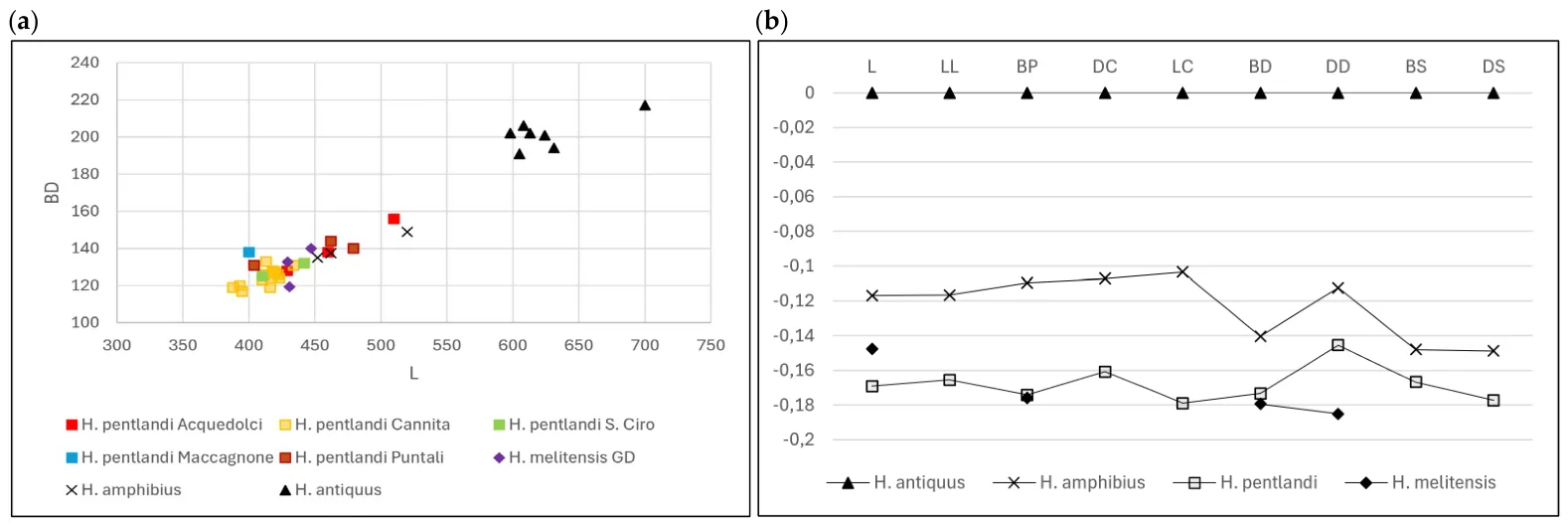
Metric comparisons of the femur: (**a**) scatterplot of BD (distal breadth) vs. L (length); (**b**) Simpson’s ratio diagram of the femur measurements, baseline: *Hippopotamus antiquus*.

In the scatterplot, *Hippopotamus melitensis* is within the range of *Hippopotamus pentlandi* (Figure 15 and Figure 16a). The Sicilian species extends from the range of *Hippopotamus amphibius* to lower values, in particular the sample from Cannita Cave. In the insular specimens, the strong reduction in length (L) is accompanied by a lesser reduction in the distal breadth (BD).

In the Simpson’s ratio diagrams (Figure 16b), the reduction in femur lengths (L, total length; LL, lateral length) of *Hippopotamus pentlandi* is almost proportional to *Hippopotamus amphibius*. The strong reduction in the femoral neck length (LC) relative to proximal breadth (BP) and head diameter (DC) quantifies the markedly shorter neck of the Sicilian remains compared to *Hippopotamus antiquus*, despite sharing the same vertical alignment.

The distal epiphysis shows a pronounced reduction in the distal breadth (BD), while the antero-posterior distal depth (DD) has a weak reduction. BD (breadth of the distal epiphysis) and DD (depth of the distal epiphisys) are reduced and parallel to *Hippopotamus amphibius*, denoting the weakly rotated trochlea, similar to *Hippopotamus amphibius*. The limited sample size available for *Hippopotamus melitensis*, does not allow significant comparisons. The tibia is stocky, with a narrow and deep intercondylar area and a tibial crest extending distally. It is more robust in the tuberosities than the more slender tibia of *Hippopotamus amphibius*.

*Hippopotamus pentlandi* shows reduction proportionally parallel to *Hippopotamus amphibius* in the lengths (L, total length; LL, lateral length) and in the proximal epiphysys breadth (BD) and depth), while the distal epiphysis breadth (BP) is near the extant species.

#### 4.4.4. Tibia

**Figure 17:**
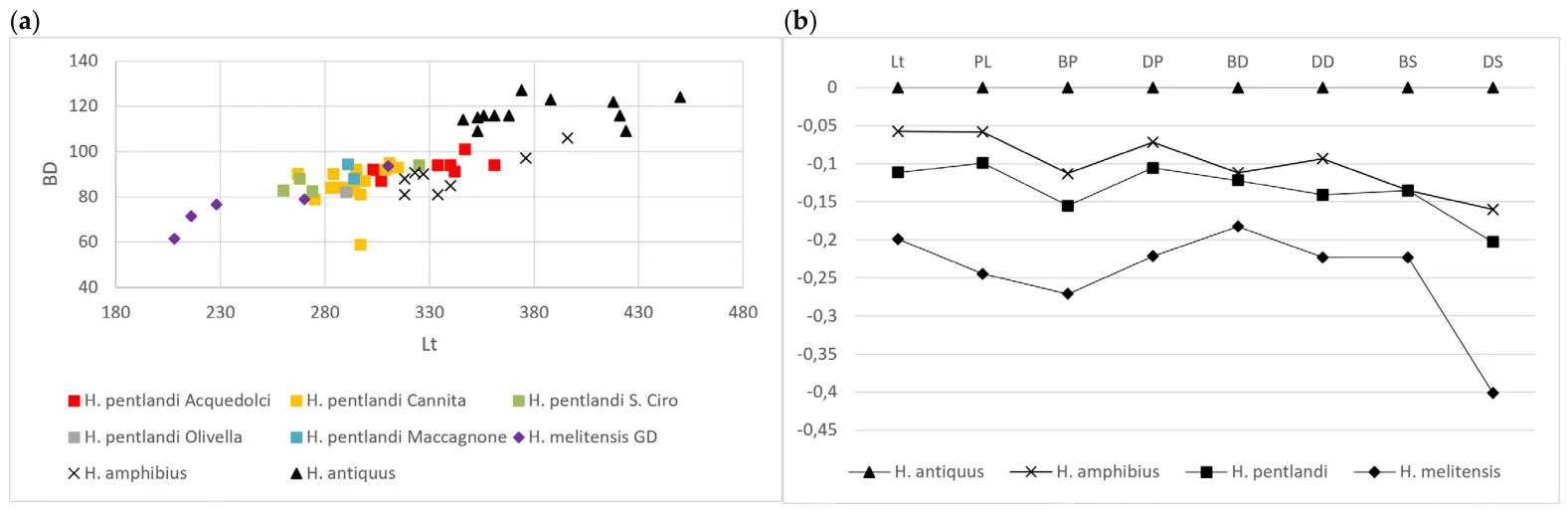
Metric comparisons of the tibia: (**a**) scatterplot of BD (distal breadth) vs. Lt (total length); (**b**) Simpson’s ratio diagram of the tibia measurements, baseline: *Hippopotamus antiquus*.

Morphologically, the tibia of *Hippopotamus melitensis* does not appear to differ from that of *Hippopotamus pentlandi*, except in size (Figure 17a). This is confirmed by the Simpson diagram, where the log-ratio profile of *Hippopotamus melitensis* runs parallel to that of *Hippopotamus pentlandi*. The Maltese tibiae have a proportionally very slender shaft (Figure 17b).

#### 4.4.5. Astragalus

The astragalus morphology of *Hippopotamus pentlandi* is characterised by a lateral trochlear lip that is more robust and more developed than the medial one, whereas in *Hippopotamus amphibius* and *Hippopotamus antiquus* the lateral and medial lips are more evenly developed, and no consistent lateral protrusion has been documented.

**Figure 18:**
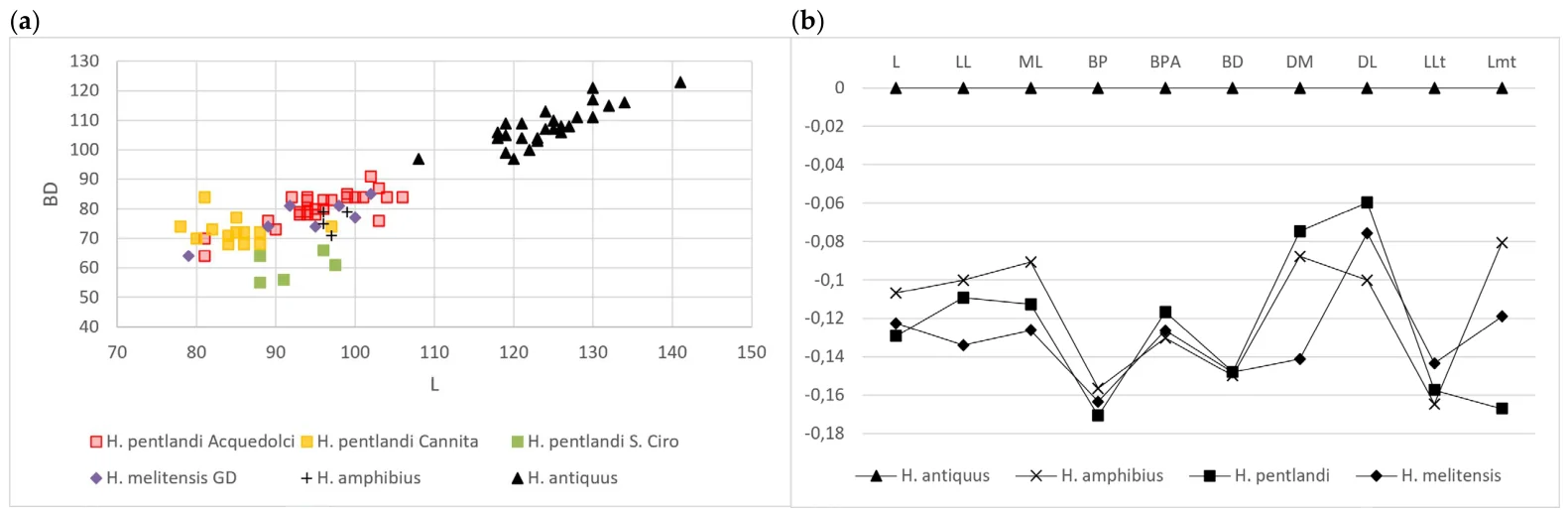
Metric comparisons of the astragalus: (**a**) scatterplot of BD (distal breadth) vs. L (total length); (**b**) Simpson’s ratio diagram of the astragalus measurements, baseline: *Hippopotamus antiquus*.

In the scatterplot of total length (L) versus distal breadth (BD) *Hippopotamus pentlandi* and *Hippopotamus melitensis* exhibit significant overlap (Figure 18a). The insular species also overlap the lower range of *Hippopotamus amphibius*, while are clearly smaller than *Hippopotamus antiquus*. Within *Hippopotamus pentlandi*, the specimens from Cannita define the lower variability, while San Ciro specimens delineate the upper variability. Astragalii from Acquedolci spans the whole variability.

The Simpson’s ratio diagram reveals a high degree of proportional alignment among the studied taxa relative to the *Hippopotamus antiquus* baseline (Figure 18b). *Hippopotamus amphibius*, *Hippopotamus pentlandi*, and *Hippopotamus melitensis* converge closely in the total length (L), proximal breadth (BP), and distal breadth (BD), where they completely converge. The distal medial depth (DM) and distal lateral depth (DL) of *Hippopotamus pentlandi* are the less reduced characters, well above *Hippopotamus amphibius*, confirming the observed robustness of the lateral throclear lip. *Hippopotamus melitensis* shows a similar trend in DL, while DM is strongly reduced. The medial trochlear length (Lmt) is reduced in *Hippopotamus amphibius* and *Hippopotamus melitensis*, whereas is strongly reduced in *Hippopotamus pentlandi*.

## 5. Discussion

The extensive Sicilian fossil record of *Hippopotamus pentlandi* contrasts with the much more fragmentary evidence available for *Hippopotamus melitensis*. Both species were subjected to intensive collecting during the 19th and early 20th centuries, lacking systematic excavations and precise geological or taphonomic constraints, besides unknown dating. Therefore, these historical museum collections of Sicily and Malta are affected by an inherent recovery bias, which is further compounded by the selective retention and exchange of specimens [12,60], introducing a degree of uncertainty. The fossil material from the Acquedolci site studied in the present paper, can be considered a reference to re-evaluate historical collections, as it originates from systematic, modern excavations [19,50,66]. The specimens were analyzed while strictly maintaining their precise provenance, thereby allowing an assessment of potential morphological or dimensional differences among localities previously noted by some authors [36,37]. Unfortunately, these conditions do not fully resolve the persistent biochronological.

The remains of *Hippopotamus pentlandi* here studied represent a sort of “brain teaser” for the co-occurrence of characters related to both possible ancestor species. Although the sample is very extensive, conclusive and unequivocal phylogenetic resolutions remain elusive. This is a problem well known in the literature, where the systematic position of *Hippopotamus pentlandi* has long been debated and mainly based on the retention of *antiquus*-like versus *amphibius*-like characters [9,10,11,16,38,58]. This already contains an important criticality, because the distinction between *Hippopotamus antiquus* and *Hippopotamus amphibius* is based mainly on combinations of cranial and mandibular characters rather than consolidated diagnostic traits [9,19,67,68]. Recently, Mecozzi et al. [22] argued that the traditional morphological differences between *Hippopotamus antiquus* and *Hippopotamus. amphibius* established in the literature [19,67] are taxonomically valid only when observed simultaneously on a single, well-preserved specimen, and may fail when applied to fragmentary material. By studying complete skull and mandible possibly of the same individual, Mecozzi et al. [22] concluded that although the specimens possess sufficient diagnostic characters to be attributed to *Hippopotamus amphibius*, a high degree of morphological overlap exists with *Hippopotamus antiquus*, rendering identifications based on isolated remains highly unreliable. Similarly, the dental morphology appears stable across the two continental species, differing primarily in size. The appendicular skeleton exhibits the same limitations.

In this study, comparisons of *Hippopotamus pentlandi* with mainland species were performed; however, the limitations identified by Mecozzi et al.ii [22], along with the conflicting data present in the literature, require a detailed and focused discussion. Based on morphology, morphometrics, and comparative analyses, the diagnostic characters of *Hippopotamus pentlandi* are here discussed, evaluating the architecture of the skeleton as a whole, rather than relying on a checklist of isolated characters.

The architecture of the head of *Hippopotamus pentlandi* can be summarized as follows. The cranium and mandible are slender, laterally compressed, and the facial region is shortened. The skull has a sturdy occipital region, with robust and horizontally oriented condyles, and flattened glenoid fossae which articulate to the correspondingly flat mandibular condyles. This architecture is completed by the narrow occipital, the wide triangular area in correspondence with the sagittal crest, and the sturdy basioccipital in the skull and the not protruding gonion caudalis, and the deep masseteric fossa in the mandible. The zygomatic arches indicate a slender skull, as well as the low horizontal ramii of the mandible. The rostral fan is not well preserved in the crania, and the metrics are contrasting for the most anterior portion, giving wider values for the mandibles, although with a reduced symphysis length. The evidence of a narrow palate with a slight constriction at M1-P4 has correspondence with the mandible. The absence of premolar diastema confirms the shortage of the face.

These characters indicate a coordinated reorganization of the cranial architecture associated with shortening and lateral compression of the skull. The reduction of the facial region was accompanied by modifications affecting both the masticatory apparatus and the occipital region. In the mandible, the combination of a slender horizontal ramus, a non-projecting gonion caudalis and a deep masseteric fossa suggests a reorganization of the masseteric musculature. Likewise, the broad triangular area associated with the sagittal crest indicates an extensive area for the insertion of the temporal musculature. The flattened mandibular condyles and corresponding glenoid fossae, together with the robust basioccipital and horizontally oriented occipital condyles, can be interpreted as complementary structural modifications associated with the shortened cranial morphology rather than as independent anatomical traits.

The upper and lower dentitions closely mirror this anatomical architecture, characterized not only by the absence of premolar diastemata but also by exhibiting teeth that are proportionally wider relative to their length. This structural trend is particularly evident in the third molars, where a decrease in length—specifically along the lingual margin—corresponds to a consistent increase in width (Figure 11b and Figure 12b: Supplementary Materials). The lower third molar significantly reduces its posterior tubercle to preserve the functional occlusal surface area despite the overall longitudinal shortening of the crown. This adjustment prevents a drastic reduction in the total occlusal surface area, which decreases proportionally less than the overall shortening of the tooth row. This trend is also maintained in *Hippopotamus melitensis*, for which cranial and mandibular remains are currently unknown.

The long bones of *Hippopotamus pentlandi* have robust shafts (SD) and wide articular epiphyses (BP, BD) despite the pronounced reduction in lengths (L, LL, PL) (Figure 13, Figure 14, Figure 15, Figure 16 and Figure 17). The astragalus also shows broad articular surfaces and a robust morphology consistent with this overall skeletal pattern that likely reflects adaptation to insular conditions.

The results presented here support an alternative interpretation to the traditional approach based on scoring individual “*amphibius*-like” and “*antiquus*-like” characters to identify the continental ancestor of *Hippopotamus pentlandi*. The anatomical and morphometric data indicate that many of these features should be interpreted within the context of the overall skeletal organization rather than evaluated as isolated taxonomic characters. Accordingly, intermediate morphologies and the coexistence of traits shared with both continental species are better interpreted as components of a coherent insular anatomical pattern than as evidence of conflicting phylogenetic affinities. Recent contributions have renewed this debate by assigning some Sicilian fossils from San Ciro Cave [69], together with the Amoroso Cave mandible [37], to *Hippopotamus amphibius*, thereby proposing the occurrence of multiple colonization events. Their interpretation is based on a combination of morphological characters and metric overlap with the mainland species. While previous contributions [9,10,11,16,38,58] provided fragmentary morphological descriptions that, through the significantly expanded sample size and observations of the present study, are here integrated into a coherent framework, Martino et al. [38] introduce a more complex scenario by proposing the presence of *Hippopotamus amphibius* in Sicily alongside *Hippopotamus pentlandi*, either as coeval taxa or through diachronic colonization events. Consequently, the focus here addresses the hypotheses and specimens analyzed by Martino et al. [38], testing whether such distinct occurrences are necessary or if the variation is better explained by an expanded dataset.

Martino et al.i [37] assigned the Amoroso Cave mandible (MandAmo1 in this paper) to *Hippopotamus amphibius* based on its robust and deep mandibular corpus, broad lateral expansion of the rostral fan, and sigmoid dental arcade, which they regarded as diagnostic of the continental species and distinct from the more slender mandibles from Cannita Cave. The Authors primarily emphasize two metric extremes of the Amoroso mandible: a short symphysis (LS = 135.1 mm), representing the lower boundary of the observed range in *Hippopotamus amphibius* (135.0–213.0 mm; mean = 177.6 mm), and a relatively high horizontal ramus (H3M = 120.7 mm). Although the latter is high for the Sicilian hippopotamus, it remains well below the mean value of *Hippopotamus amphibius* (146.8 mm; 48 specimens, Martino dataset). A similarly high horizontal ramus is also recorded in *Hippopotamus pentlandi* from Serraci (MandSer1; H3M = 127 mm; this study). Some discrepancies between the measurements reported by Martino et al. [37] and those of this paper are most likely related to differences in the application of measurement protocols and/or the extensive museum restoration of the specimen (Table A2). Despite these differences, both datasets fall entirely within the range of *Hippopotamus pentlandi* (Figure 10) and remain substantially lower than the corresponding mean values of *Hippopotamus amphibius* (177.6 mm and 385.0 mm, respectively, calculated on the Martino dataset). A further discrepancy concerns the external breadth of the rostral fan and can be explained by differences in landmark selection or by alternative interpretations of the deformation introduced by the extensive restoration. The lower third molars (MandAmo1s and MandAmo1d, this paper) display a reduced posterior tubercle (M/3Lpt = 17.0 and 19.0 mm), corresponding to approximately 26% of tooth length (Lpt × 100/OL). This value is intermediate between the Acquedolci sample (approximately 30%) and the Cannita sample (approximately 23%), whereas both continental species average about 32%. In conclusion, the present morphological and morphometric evidence does not favor assigning the Amoroso mandible to *Hippopotamus amphibius*, as its characters are fully compatible with the broader *amphibius*-like pattern observed in *Hippopotamus pentlandi*. Martino et al [70] identified some postcranial specimens from the San Ciro Cave collection as falling within the morphological and metric variability of *Hippopotamus amphibius*, including humeri (SC67: fragmented left humerus exhibiting a fairly preserved distal epiphysis and a partially preserved deltoid tuberosity; SC68, right distal humerus fragment with a fairly preserved distal epiphysis; SC331 left distal humerus fragment), a radio-ulna (SC70, right radio-ulna with fused radius and ulna), a femur (SC80, complete, but broken into two distinct portions), tibiae (SC87 and SC88, complete), and a third metatarsal (SC113b). Most of these specimens are included in the original dataset of the present study (Supplementary Materials).

The authors assigned the humeri to *Hippopotamus amphibius* by the overall dimensions consistent with the modern species (the medial distal depth, DDm, in MGUP SC68), combined with a prominent distal development of the medial epicondyle and the absence of a heavily robust lateral epicondyle, which they consider characteristic of *Hippopotamus pentlandi*. The measures (DDm, medial, and DDl, lateral) support this description. However, when expanding the set of comparative measurements to include transverse dimensions and extending the reference sample to a broader sample, the attribution of the humeri to the continental species is questionable (Figure 13 and Figure 14). Martino et al. [69] argue that the DDm of SC68 (129.5 in their dataset; 128 mm in the present paper) falls within the *Hippopotamus amphibius* range. However, the data provided in this paper indicate that it falls within the sample from Acquedolci, which reaches significantly higher distal depths, such as OmAcq2 (130 mm) and OmAcq6 (135 mm) (Figure 13 and Figure 14, Supplementary Materials). Moreover, including the breadth metrics not considered by Martino et al. [70], specimen Om SC2 (SC67) exhibits a proximal breadth (BP = 136 mm) completely congruent with the *Hippopotamus pentlandi* range from Acquedolci (122–154 mm) and Cannita (110–150 mm) (Figure 13 and Figure 14). Similarly, for Om SC4 (SC68), the distal breadth (BD = 128 mm) and minimum shaft width (BS = 57.5 mm) are within the known variability of the Sicilian specimens. Furthermore, the inclusion in the present paper of Om SC3 (SC67 bis), with a BD of 115 mm and a DDm of 120, documents a continuous dimensional transition within the San Ciro assemblage rather than a dimensional break. Finally, the strong distal epiphyses are present in very sturdy humeri, and the prominence of the medial epicondyle can be related to robust flexor muscles in a very large individual, more than a taxonomic character.

The radius-ulna SC70 (RuSC1 in this paper, Supplementary Materials) is referred to *Hippopotamus amphibius* by Martino et al. [69] based on the wide angle between the long axis of the olecranon and the radial shaft, combined with a robust radius and a robust olecranon. By a dimensional point of view, the specimens plot with the lower range of *Hippopotamus amphibius*, but also with the range of *Hippopotamus pentlandi* (this paper, Supplementary file). The RI is calculated in the excel sheet using the formula (BSr ∗ π)/Lr) ∗ 100, without additional explanation in the text. The recalculated RI applying the standard formula (BSr/Lr) ∗ 100 on the Martino et al. [69] database gives the following values: SC70 RI = 16; *Hippopotamus amphibius* RI = 16.; *Hippopotamus pentlandi* RI = 20. The RI calculated on this paper dataset for specimens from Acquedolci and Cannita is 19. SC70 is effectively comparable in slenderness to *Hippopotamus amphibius*. However, in the scatterplot of BPr (proximal breadth of the radius) vs. Lr (radius length) (Figure 15a), the point representing SC70 (the San Ciro’s outlier visible in Figure 15a) deviates from both *Hippopotamus pentlandi* and *Hippopotamus amphibius*, shifting toward an extremely slender morphology. This evidence likely reflects an individual variation in marked slenderness.

The femur SC80 (FemSC4 in this paper, Supplementary Materials) is described as robust, with massive epiphyses and wide diaphysis and assigned to *Hippopotamus amphibius* based on its robust neck and the well-marked and relatively deep supracondylar and intercondylar fossae. However, the broad and markedly short femoral neck, the robust shaft, and the greater trochanter that reaches approximately the same height as the femoral head are common with the *Hippopotamus pentlandi* characters described in this paper. The RI calculated by Martino et al. [70] for SC70 is 14.78, the other San Ciro specimens of their dataset have RI = 14.98 (SC77) and 12,89 (SC79), while *Hippopotamus amphibius* has a mean RI = 12.5. Therefore, the remarkable length of SC80 is not accompanied by the slenderness of the femur typical of *Hippopotamus amphibius*, but it is fully consistent with the proportional sturdiness of the Sicilian species. Moreover, although the length of SC80 has not been measured in this paper due to potential measurement bias introduced by the fracture that divides the bone in two halves, its length (L = 448 mm, Martino et al. [69]), is not anomalous when integrated into a wider dataset. Specimens from other sites are longer, such as two femurs from Acquedolci (FemAcq1 = 510 mm; FemAcq2 = 460 mm) and Puntali (FemPun2 = 462 mm; FemPun3 = 479 mm). The lengths of the Puntali specimens previously led Capasso Barbato and Petronio [36] to infer the possible presence of the continental species in Sicily. Data from Acquedolci, spanning to a wide range, confirm the congruence with the Sicilian species.

In conclusion, the assignments proposed by Martino et al. [70] rely on selected appendicular features and local morphometric variables. As discussed above, the expanded comparative dataset presented here shows that these characters fall within the observed variability of *Hippopotamus pentlandi* when evaluated within the broader anatomical variability of the species. Moreover, Martino et al. [69] themselves acknowledge that the overall robustness and shortening of the limbs, traditionally regarded as characteristic of *Hippopotamus pentlandi*, are not exclusively diagnostic but largely reflect adaptation to insular conditions. This interpretation is consistent with the anatomical pattern recognized in the present study, while substantially reducing the taxonomic significance of the appendicular characters used to support the attribution of isolated Sicilian specimens to *Hippopotamus amphibius*. Furthermore, caution is required, as subtle shape shifts or isolated metric overlaps extracted from restricted 3D and multivariate datasets risk overinterpreting localized allometric variation for true taxonomic heterogeneity.

Finally, the interpretation of *Hippopotamus pentlandi* as a predominantly terrestrial grazer, characterized by a slender skull associated with short, robust limbs, is further supported by the dental microwear analyses of Martino et al. [70]. Their results show a significantly higher pit count in *Hippopotamus pentlandi* than in *Hippopotamus amphibius*, *Hippopotamus antiquus* and *Hippopotamus gorgops*. This pattern indicates a grazing diet with substantial ingestion of exogenous dust and grit, suggesting occupation of more open and arid environments than those inhabited by the extant hippopotamus. The absence of puncture pits further argues against the regular consumption of hard fruits and is consistent with a predominantly terrestrial grazing ecology.

The discussion regarding the results for *Hippopotamus melitensis* is more difficult due to the scarcity of available remains which does not allow assessment of whether *Hippopotamus pentlandi* was also present on the island. To avoid unnecessary taxonomic ambiguity, the entire Maltese record is herein referred to as *Hippopotamus melitensis*. Nevertheless, highly significant data are provided by the third upper and lower molars, which show morphological patterns similar to *Hippopotamus pentlandi*. The sample of lower third molars, integrated with the numerous data from van Huut [58], exhibits a remarkably wide range of variation for the Maltese species, which continuously overlaps with the variability of the Sicilian taxon (Figure 12a). The size distribution concentrates around intermediate values aligning with the “bell-shaped” distribution by van Huut [58], attributed by this Author to the wide intraspecific variability of *Hippopotamus melitensis* rather than to the presence of two species. However, a small cluster of molars reduced in size is observable in the upper (Figure 11a) and lower (Figure 12a) molars. In the upper molars, also an outlier of Sicily (San Ciro cave) falls in the strongly reduced cluster (Figure 11a. The Maltese upper molars do not further reduce the inner length (IL) with the respect to *Hippopotamus pentlandi* (Figure 11b), while the trend toward a reduced posterior tubercle is enhanced (Figure 12b). Regarding the postcranial remains, the few specimens, some of them fragmented, precludes definitive conclusions. The radius-ulna and the femur are within the variability of *Hippopotamus pentlandi* (Figure 15 and Figure 16, respectively), whereas the humerus and the tibia fall within the range of the Sicilian species (Figure 13 and Figure 14 and Figure 17, respectively). The astragalus exhibits a morphology consistent with *Hippopotamus pentlandi* and dimensionally overlaps with the Sicilian species (Figure 18). Also in the case of Maltese fossils, the provenance of the material from historical collections remains unresolved due to the lack of precise stratigraphic context. While the data provided in this study are insufficient to definitively settle the debate on Maltese hippopotamuses, they nonetheless highlight a close relationship with the Sicilian lineages.

Finally, a formal taxonomic revision lies beyond the scope of the present study. To maintain clarity and avoid unnecessary nomenclatural confusion, the Sicilian material is herein referred to *Hippopotamus pentlandi* and the Maltese record to *Hippopotamus melitensis*, in accordance with prevailing nomenclature.

## 6. Conclusions

The direct examination of historical collections together with newly recovered material, including the systematically excavated Acquedolci assemblage, considerably expands the anatomical and morphometric documentation of *Hippopotamus pentlandi*. The enlarged comparative sample shows continuous morphological variation among the Sicilian populations, with no morphological or morphometric discontinuities supporting the recognition of more than one hippopotamus taxon within the material examined.

Comparison with the continental species confirms the mosaic of characters that has long complicated the identification of the ancestral taxon of *Hippopotamus pentlandi*. At the same time, the Sicilian material documents a distinctive skeletal organization combining a shortened, more gracile skull with shortened, robust limbs. These features do not represent an assemblage of isolated characters shared with either *Hippopotamus antiquus* or *Hippopotamus amphibius* but form a coherent anatomical pattern that characterizes the endemic Sicilian species.

The expanded morphometric dataset substantially reduces the morphological separation previously proposed among Sicilian hippopotamus populations. The Acquedolci assemblage bridges much of the apparent gap between the historical collections, showing that large specimens, including the Amoroso mandible and several long bones from San Ciro Cave, fall within the variability of *Hippopotamus pentlandi*, although they approach the lower limit of variation of *Hippopotamus amphibius*. Consequently, the material currently available does not support the recognition of additional hippopotamus taxa in Sicily.

The Maltese material overlaps the morphology and the lower morphometric range of *Hippopotamus pentlandi* while showing a general trend towards further size reduction. However, the available record is still insufficient to characterize *Hippopotamus melitensis* adequately, and the occurrence of *Hippopotamus pentlandi* on in Malta cannot presently be excluded.

The anatomical evidence indicates that the evolution of the Sicilian hippopotamus involved coordinated modifications of both the cranial and appendicular skeleton rather than body-size reduction alone. The subsequent history of the Maltese populations remains poorly documented because of the fragmentary fossil record. The anatomical evidence presented here provides a solid basis for future phylogenetic analyses of the Mediterranean insular hippopotamuses.

## Data Availability

Data is contained within the article or Supplementary Materials.

## Supplementary Materials

The following supporting information can be downloaded at: https://www.mdpi.com/article/10.3390/life16081276/s1.

## Abbreviations

The following abbreviations are used in this manuscript:

M3, M2, M1	Upper third, second and first molars
P4, P3, P2	Upper fourth, third, and second premolars
C	Upper canine
I2, I1	Upper second and first incisors
m3, m2, m1	Lower third, second and first molars
p4, p3, p2	Lower fourth, third, and second premolars
c	Lower canine
i2, i1	Lower second and first incisors

## Appendix A

State of preservation of the studied crania (Table A1) and mandibles (Table A2) of *Hippopotamus pentlandi*.

**Table A1:** State of preservation and age classes of the studied crania of *Hippopotamus pentlandi*, divided by site and ordered by age classes. Age classes: subadult, mixed deciduous and permanent dentition; adult, permanent dentition fully erupted; old, permanent dentition fully erupted and heavily worn. Repository, original inventory numbers and other details associated with the specimen’s label are in S1.

Site	Specimen	State of Preservation	Age Class
Acquedolci	CrAcq1	Specimen affected by numerous fractures. Neurocranium and zygomatic arches largely preserved; rostral region largely missing; occipital region well preserved. Cheek teeth preserved from P3 to erupting M3 (in the alveolus); D4 retained between M1 and P3	subadult
	CrAcq2	Fragmentary palatine process of the maxilla with left M1–M3 and right M2–M3 well preserved	adult
	CrAcq3	Heavily fragmented, with a mosaic of slightly displaced fragments; plastically deformed; anterior rostral region missing. Orbits broken at their bases and displaced ventrally; collapsed sagittal crests; one canine dislocated into the nasal cavity. Cheek dentition well preserved from P2 to M3.	adult/old
	CrAcq4	Central portion of the skull preserved; neurocranium and rostrum missing; intensely fragmented, with slight displacement among fragments and marked deformation; cheek tooth rows partially preserved (M1 missing on both sides; left P2 absent).	old
	CrAcq5	Dorsal portion relatively well preserved, with slight displacement along fragmentation; anterior rostrum missing; zygomatic arches intact; occipital region well preserved; ventral portion poorly preserved; cheek tooth rows partially preserved; both M1 and leftP4 absent.	very old
	CrAcq6	Occipital fragment with a small portion of the occipital squama, occipital condyles, basioccipital, and glenoid fossae preserved.	---
	CrAcq7	Fragmentary neurocranium with partially preserved occipital.	---
	CrAcq8	Fragmentary occipital	---
	CrAcq9	Fragmentary occipital.	---
Cannita Cave	CrCan1	Concretion-covered, deformed, and dorsoventrally compressed fragment; preserving a premolar in alveolus, D4, M1, M2, M3 in alveolus, and a small canine.	juvenile
	CrCan2	Fragment preserving the right half of the occipital squama, parietals, sagittal crest, temporal lines, and part of the frontal, right orbit ventrally displaced; heavily damaged alveolar portion with a M2 and M3 in the alveolus.	juvenile/subadult
	CrCan3	Heavily deformed and largely reconstructed specimen; only the palate is observable, preserving teeth from P3 to M3, some displaced from their physiological position; left I2 and left canine preserved.	adult
	CrCan4	Heavily reconstructed maxillary fragment preserving the left dentition from I2 to M2 and the right dentition from C to P3.	adult/old
	CrCan5	Deformed fragment, preserving the frontal, nasals, and palate; portion anterior to P2 and nuchal region missing. Dentition preserved from P2 to M3.	old
	CrCan6	Maxillary fragment preserving the right dentition from P3 to M3 and the left dentition from M1 to M3.	old
	CrCan7	Heavily compressed and deformed, especially in the frontal and anterior regions; left zygomatic arch, left glenoid cavity, occipital squama, and part of the sagittal crest relatively well preserved: right M1–M3 preserved.	very old
	CrCan8	Neurocranial fragment with occipital condyles preserved.	---
Malatacca Cave	CrMal1	Restored, partially concretion-covered right maxillary fragment preserving M2 and M3.	adult
	CrMal2	Partially concretion-covered left maxillary fragment preserving M1.	juvenile
Maccagnone Cave	CrMac1	Maxillary fragment preserving M2 and M3; M1 broken.	old
San Ciro Cave	CrSc1	Left palatal fragment preserving M1–M2, M3 in the alveolus; remnants of attached breccia.	juvenile
	CrSc2	Left maxillary fragment with M2 and M3.	subadult
	CrSc3	Left maxillary fragment with M2.	adult
	CrSc4	Left maxillary fragment with P4-M2.	adult
	CrSc5	Left maxillary fragment with M2 and M3.	old
	CrSc6	Maxillary fragment preserving the left M2 and M3 and the right M2.	adult/old
	CrSc7	Palatal fragment with alveolar portion preserved; right M2 and M3 preserved.	old
	CrSc8	Occipital fragment preserving the left glenoid fossa, basioccipital region, and part of the braincase; fossilization pattern distinct from that observed in other specimens from the same locality.	---
	CrSc9	Fragmentary palate without teeth.	---
	CrSc10	Palatal fragment lacking dentition.	---
	CrSc11	Fragment preserving portions of the frontal and nasals; palate well preserved.	---
	CrSc12	Fragmentary occipital fragment lacking the occipital condyles; portion of the parietal preserved, sagittal crest broken.	---
	CrSc13	Occipital fragment with frontal and the sagittal crest preserved.	---
	CrSc14	Occipital fragment with frontal and the sagittal crest preserved.	---
	CrSc15	Occipital fragment with parietal and the sagittal crest preserved.	---
	CrSc16	Small (juvenile?) occipital fragment	---
Serraci		Well-preserved neurocranium with the occipital region, zygomatic arches, orbits, and part of the nasals preserved. Dentition and palate not preserved.	---

**Table A2:** State of preservation and age classes of the studied mandibles of *Hippopotamus pentlandi*, divided by site and ordered by age classes. Age classes: subadult, mixed deciduous and permanent dentition; adult, permanent dentition fully erupted; old, permanent dentition fully erupted and heavily worn; stages according to Laws, 1968. Repository, original inventory numbers and other details associated with the specimen’s label are in S1.

Site	Specimen	State of Preservation	Age Class
Acquedolci	MandAcq1	Left mandible fragment with M1 and M2.	juvenile
	MandAcq2	Mandible with the anterior portion and the upper part of the ascending ramus missing. Cheek dentition preserved bilaterally, except for M1.	subadult Laws stage XI
	MandAcq 3	Nearly complete mandible, with localized fragmentation. Only the coronoid and condylar processes, together with the ventral margins of the ascending rami, are missing. Complete dentition preserved.	adult Laws stage XIV
	MandAcq4	Heavily fragmented and deformed mandible, with recementation along fractures. Cheek teeth well preserved, from left P4 to M3 and right M2 to M3. Both I2 preserved; canines fragmented and recemented.	adult Laws stage XVI
	MandAcq5	Mandible lacking most of the symphyseal region, the ascending rami, and the ventral portions of the horizontal rami. Possible paleopathology indicated by the presence of exposed cancellous bone over a large area of the left horizontal ramus. Dentition preserved from P2 to M3 on the right side; left P2, M1, M2, and M3 preserved.	old Laws stage XVII
	MandAcq6	fragment of left horizontal ramus with P3, M2 and M3.	old Laws stage XVII
	MandAcq7	fragment of right horizontal ramus with M3 the roots of M2.	very old
	MandAcq8	fragment of right horizontal ramus with M2 and M3	very old
	MandAcq9	fragment of the anterior portion with P2, C, I1, and I2	adult
Amoroso cave	MandAmo1	mandible lacking ascending ramus, with a strongly restored inferior border of the horizontal ramus; pervasive museological restoration.	adult Laws stage XIV
Cannita Cave	MandCan1	Left hemimandible with complete ascending ramus and anteriorly incomplete horizontal ramus; M1–M3 preserved.	adult Laws stage XIV
	MandCan2	Right hemimandible with restored ascending ramus; P3-M3 preserved.	adult Laws stage XV
	MandCan3	Fragment of the right horizontal ramus preserving P3, P4, M2, and M3.	adult Laws stage XVII?
San Ciro Cave	MandSc1	Fragmentary left ascending ramus; all teeth broken at the roots except M3.	
	MandSc2	Fragment of horizontal ramus with M3	adult
	MandSc3	Anterior mandibular fragment.	
	MandSc4	Fragment of posterior portion	---
	MandSc5	Horizontal rami partially preserved, with broken ventral margins. Only the right I2 preserved; all remaining teeth broken at the roots.	---
	MandSc6	Anterior mandibular fragment.	
	MandSc7	Anterior mandibular fragment preserving the symphysis.	---
	MandSc8	Anterior mandibular fragment preserving the symphysis.	---
	MandSc9	Anterior mandibular fragment preserving the symphysis.	---
	MandSc10	Anterior mandibular fragment preserving the symphysis.	---
	MandSc11	Anterior mandibular fragment preserving the symphysis.	---
Serraci	MandSer1	Posterior portion, preserving the horizontal ramus under m2-m3, the angle. The ascending ramus is not preserved. Strong concretion.	
